# Design of a Novel Low Cost Point of Care Tampon (POCkeT) Colposcope for Use in Resource Limited Settings

**DOI:** 10.1371/journal.pone.0135869

**Published:** 2015-09-02

**Authors:** Christopher T. Lam, Marlee S. Krieger, Jennifer E. Gallagher, Betsy Asma, Lisa C. Muasher, John W. Schmitt, Nimmi Ramanujam

**Affiliations:** 1 Department of Biomedical Engineering, Duke University, Durham, North Carolina, United States of America; 2 Department of Surgery, Duke University, Durham, North Carolina, United States of America; 3 Center for Global Women’s Health Technologies, Duke University, Durham, North Carolina, United States of America; 4 Duke Global Health Institute, Duke University, Durham, North Carolina, United States of America; 5 Department of Obstetrics and Gynecology, Duke University, Durham, North Carolina, United States of America; ACTREC, Tata Memorial Centre, INDIA

## Abstract

**Introduction:**

Current guidelines by WHO for cervical cancer screening in low- and middle-income countries involves visual inspection with acetic acid (VIA) of the cervix, followed by treatment during the same visit or a subsequent visit with cryotherapy if a suspicious lesion is found. Implementation of these guidelines is hampered by a lack of: trained health workers, reliable technology, and access to screening facilities. A low cost ultra-portable Point of Care Tampon based digital colposcope (POCkeT Colposcope) for use at the community level setting, which has the unique form factor of a tampon, can be inserted into the vagina to capture images of the cervix, which are on par with that of a state of the art colposcope, at a fraction of the cost. A repository of images to be compiled that can be used to empower front line workers to become more effective through virtual dynamic training. By task shifting to the community setting, this technology could potentially provide significantly greater cervical screening access to where the most vulnerable women live. The POCkeT Colposcope’s concentric LED ring provides comparable white and green field illumination at a fraction of the electrical power required in commercial colposcopes. Evaluation with standard optical imaging targets to assess the POCkeT Colposcope against the state of the art digital colposcope and other VIAM technologies.

**Results:**

Our POCkeT Colposcope has comparable resolving power, color reproduction accuracy, minimal lens distortion, and illumination when compared to commercially available colposcopes. *In vitro* and pilot *in vivo* imaging results are promising with our POCkeT Colposcope capturing comparable quality images to commercial systems.

**Conclusion:**

The POCkeT Colposcope is capable of capturing images suitable for cervical lesion analysis. Our portable low cost system could potentially increase access to cervical cancer screening in limited resource settings through task shifting to community health workers.

## Introduction

Invasive cervical cancer (ICC) affects the lives of 500,000 women worldwide each year, and results in more than 270,000 deaths [1]. More than 75% of ICCs occurs in Africa and India, with the highest incidence occurring in East Africa [2]. ICC is highly preventable by treating its precursor lesions and early stage cancers. However, these prevention services are not widely available in many low and middle-income countries (LMICs). Pap smear-based screening and HPV testing, which are widely available in western countries have not been feasible to implement widely in LMICs owing to their cost, lack of infrastructure and appropriately trained human resources. Randomized controlled trials conducted in India and other LMICs have shown that visual inspection with acetic acid (VIA) is the most resource-efficient approach to screen for cervical cancer [3]. VIA is just a simpler version of colposcopy which is used in western countries to diagnose cervical pre-cancer/cancer in women who have already been screened and found to have a positive Pap smear [4]. During colposcopy, the cervix is exposed using a speculum and visualized at low magnification (4-7X) for subtle features on the cervix [5, 6]. Whitening of the cervix from the application of acetic acid is use to determine the presence of lesions, through visualization of heterogeneity of suspicious regions (opacity, color, shape, pattern). A green filter can be applied to the colposcope’s illumination source to aid in the visualization of vascularization, a known hallmark of severe dysplasia [5, 6]. The prohibitive cost of colposcopes (US$ 10,000–20,000) limits their uptake in resource-limited settings [7, 8]. Therefore, VIA is performed with a simple headlamp in most instances.

VIA has several implementation challenges that limit the scale and hence, impact of this approach (1). Challenges include: 1) high screen positivity rates (likely due to inadequate training and technology), leading to a high volume of patients being referred for follow up and at the same time, a very high loss to follow-up (about 50%) of the large number of screen-positive women [9]. If community health workers could be empowered to bring colposcopy to the primary care setting and be trained to more effectively use this technology to triage women, secondary and tertiary care facilities could focus their energies on managing priority patients who are in most urgent need of follow-up care, without being overwhelmed by women who don’t need treatment.

Prior attempts to develop and implement low cost colposcopes for use in limited resource settings have met with some success. These include a low-cost hand-held portable analog colposcope (Aviscope) [10–14], the Magnivisualizer [15–17], and the Family Health Ministries-Duke Portable Colposcope, which is based on surgical loupes. Prior low cost colposcope devices have had limited success stemming from: lack of digital image capture capability, fixed magnification, limited depth of focus, and poor illumination characteristics, [10–14]. A more recent system (Gynocular) has addressed some of these issues with the potential to have digital colposcopy capabilities [18, 19].

We have developed a novel, low cost Point of Care Tampon digital Colposcope (POCkeT Colposcope) to address many of the limitations described above for low cost colposcopes. The POCkeT Colposcope is shaped like a tampon and can be inserted and positioned such that it is 30–40 mm away from the cervix, obviating the need for high-end optics and high-resolution cameras used in state of the art colposcopes, which need to have a working distance of 300 mm. In fact, our POCkeT Colposcope leverages consumer grade light sources and cameras used in smart phones and 3D printing is used to create the tampon form factor. The POCkeT Colposcope’s images are comparable to that of a state-of-the-art digital colposcope by virtue of the fact that it is placed inside the vagina like a trans-vaginal ultrasound probe at a much closer working distance than a traditional colposcope. Frontline health workers trained to conduct VIA should be able to deploy the POCkeT Colposcope and obtain colposcopy images that will duplicate what is achievable with state of the art colposcopy. An Android operating system based phone/tablet coupled via the OTG (on-the-go) USB (universal serial bus) communication protocol to the device allows digital images to be transmitted to tertiary centers and reviewed by expert physicians so that referrals are made for only those women who require treatment. The images along with the health worker and physician diagnosis and when available, confirmatory biopsy can be uploaded to a database that can eventually be utilized as a virtual training tool for dynamic improvements in quality control and improve the quality of training of future health workers. Data can also be archived for retrospective or longitudinal studies.

As HPV testing becomes more prevalent there is promise for wide spread screening for cervical cancer. However, VIA will still be a bottleneck in the recommended “see and treat” paradigm. A low cost ultra-portable digital colposcope system is essential to triage women and/or follow women who are HPV positive at the community level setting. Digital capture of high quality images can allow for a repository of images to be compiled that can be used to empower front line workers to become more effective through virtual and dynamic training. By shifting high-level care typically practiced at the secondary and tertiary levels to a community setting, this technology will provide significantly greater cervical screening access to the communities where the most vulnerable women live.

## Materials and Methods

### System design

**Fig 1:**
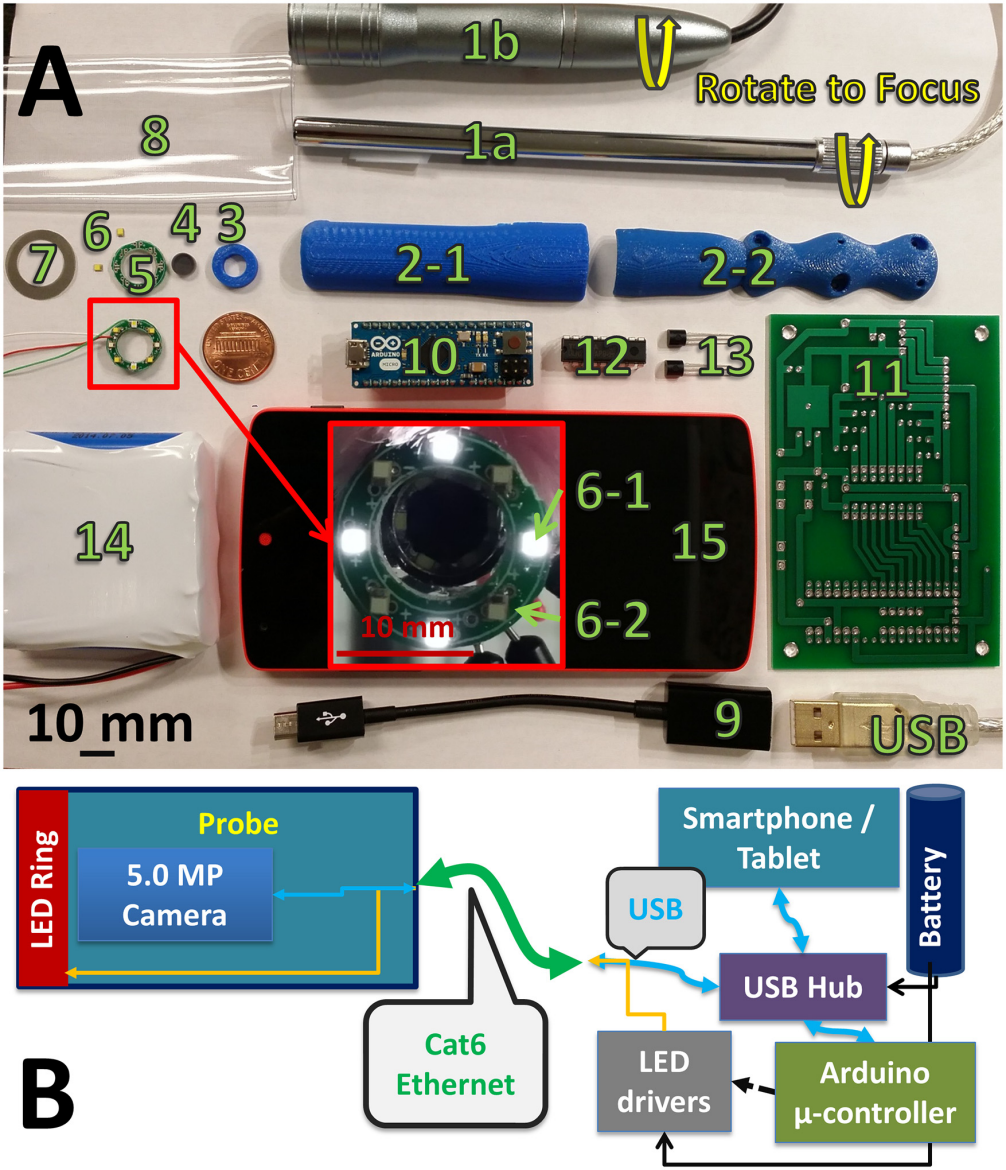
System schematic of our Point of Care Tampon Colposcope (POCkeT Colposcope) with computer aided 3D rendering. (A) of the assembled device with the essential system components (1) USB cable, (2) manual focus knob, (3) probe handle or body, (4,7) clear sterile disposable sheath, (5) ABS 3D printed shell, (6) linear glass polarizer; and 3D rendering (B) of the exploded schematic view of the device (8) the color CMOS detector, (9) clam shell ABS probe handle, (10) polarizer and illumination ring holder, (11) linear glass polarizer, (12) concentric white and green LED ring, (13) linear film polarizer, (14) clear sterile disposable sheath; and a block diagram (C) of the system interface that combines a single communication cable (Cat5e) to allow for 2-way communication and control of the camera and LED ring through the Smartphone/Tablet and Arduino Microcontroller via a powered USB hub.

The components of the POCkeT Colposcope are designed to fit within the form factor of a tampon, with an outer diameter of 20 mm and length of approximately 140 mm. Either a 2 or 5 MP (megapixel) color CMOS camera uses plastic lenses with 3 to 5 lens elements in a prime lens configuration with an infinity focus, and a back focal length of between 3 to 4 mm. The f/# is between 2 and 3, with emphasis for wide angle imaging [20]. The POCkeT Colposcope has a concentric illumination ring with white and green LEDs to mimic the functionality of traditional colposcopes, and is cross-polarized to minimize specular reflection [21–24]. Fig 1, is a exploded view schematic of the system: (1) USB 2.0 interface, (2) rotational adjustment, (3) base of the digital applicator, (4) pliable tampon like introducer tip, (5,8) 2 to 5 MP color CMPS auto-focus imager, (6) hydrophobic optical window, (7,14) clear disposable sterile sleeve barrier to minimize contamination of probe, (9) injection molded ergonomic probe outer shell, (10) polarizer and illumination ring holder, (11) glass linear polarizer, (12) white and green LED concentric illumination ring and (13) thin film linear polarizer. An Android device interfaces with an Arduino microcontroller controller that controls the constant current LED drivers and subsequently the intensity of the LEDs with Pulse Width Modulation (PWM) signals.

### Characterization of Imaging System

We set out to quantitatively assess the imaging performance of the POCkeT Colposcope and standard colposcopes, as well as other illumination/imaging systems used for VIA with magnification. These systems range from an unmodified smartphone approach to a high-end Leisegang Optik 2 digital colposcope (Cooper Surgical Inc., Trumbull CT). The commercial system is a Leisegang Optik 2 that integrates an off the shelf DSLR camera (a Canon Rebel T3i, 18MP CMOS) for digital image capture and has 3 selectable magnifications (3.75, 7.5, and 15 X). A second commercial system is a Wallach Tristar (Wallach Surgical, Orange CT) without digital image capture capability. It has continuous magnification from 7 to 30X. Our POCkeT Colposcope systems use either a 2.0MP color CMOS sensor with manual focus or a 5.0MP color CMOS sensor with automatic focus mechanism. In resource limited settings, some clinics have selected to use off-the-shelf consumer devices (Canon SX50HS super zoom digital camera and unmodified Apple iPhone 5S) which were also included in the comparison. There exists a significant difference in cost, weight, and volume when comparing our POCkeT Colposcope system to the other implementations currently in use (Table 1). The reason that the other low cost colposcopes described in the introduction were not included was because they do not have integrated digital image capture capability.

**Table 1:** Overview of a specifications of a digital colposcope systems.

System	Price (US$)	Weight (lbs)	Clinical Sites	Light Source	Detector Type	Resolution (MP)	Pixel Pitch (μm)	Sensor Size	Aspect Ratio	Focus
Leisegang Optik 2	20,000	175	DUMC	18W LED, LP Green Filter	CMOS	18 (5184x3456)	4.3	APS-C	3:2	Manual
Wallach Tristar	6,500	75	KCMC*	150W Halogen, LP Green Filter	Not Applicable					
Canon SX50HS	399	1.3	KCMC*	Uses Wallach Tristar source	CMOS	12.1 (4000x3000)	1.5	1/2.3”	4:3	Auto
Apple iPhone 5S	800	0.25	CACHA	White LED Flash	CMOS	8.0 (3264x2448)	1.5	1/3.0”	4:3	Auto
2.0MP POCkeT Colposcope	300	0.75	DUMC	1W White LEDs	CMOS	2.0 (1600x1200)	2.2	1/4"	4:3	Manual
5.0MP POCkeT Colposcope	300	1.0	DUMC	2W White and Green LEDs	CMOS	5.0 (2592x1944)	1.4	1/4"	4:3	Auto

High-end digital colposcope (Leisegang Optik 2), a traditional colposcopes (Wallach Tristar), and digital cervicography devices (Canon SX50HS and Apple iPhone 5S) with respect to cost, weight, clinical sites, and light source strategies when compared to the novel 2.0MP (megapixel) and 5.0MP POCkeT Colposcope Clinical sites include: Duke University Medical Center (DUMC) in Durham, NC; Kilimanjaro Christine Medical Center (KCMC) in Moshi, Tanzania; and Canada Africa Community Health Alliance (CACHA) in Moshi, Tanzania.

* For digital cervicography at KCMC, the Canon SX50HS digital camera uses the halogen light source of an analog colposcope. APS-C stands for Advanced Photo System type-C imager format.

### Characterization of Digital imaging quality

There is no currently internationally recognized standard methodology for assessing imaging performance of digital colposcopes [25]. Prior groups have reported a battery of targets and tests for performance assessment and/or digital colposcope calibration [26–28]. We first set out to confirm the reported imaging specifications of these different systems including: resolving power (micron), working distance (mm), and diagonal field of view (mm), at a range of possible magnifications, if the system was capable of multiple magnifications. Magnification of each system was confirmed using an USAF 1951 resolution target to determine the minimal resolvable line width (micron), and diagonal field of view using a 2x2 mm square checkerboard grid. The smallest resolvable element and group was used to determine the resolving power, from measurements by a pair of independent observers for each system.

Radial Lens Distortion was characterized using a checkerboard grid and the percentage distortion was calculated using the Standard Mobile Imaging Architecture (SMIA) TV Distortion formula as a percentage of barrel or pincushion distortion [25]. Undistorted squares will have distortion equal to 0%, while positive distortion is define as pincushion or inward bowing of the image and negative distortion is defined as barrel or outward swelling of the image. Percentage distortion is equal to $\frac{100*(A-B)}{B}$, and A is equal to $\frac{A_{2}-A_{1}}{2}$, where ***A***_***1***_ and ***A***_***2***_ are the outer side lengths of a square and ***B*** is the distance between the midpoints of the sides of the square ***A***_***1***_ and ***A***_***2***_ [29]. This type of distortion is of potential concern as the pixel to length ratio is no longer constant and size and shape of structures will be distorted more at the edges of the image when compare to the center and could affect colposcopic interpretation where symmetry or lack thereof are potential hallmarks of cervical disease.

The color accuracy of each digital system was characterized by imaging an NIST-calibrated color target (X-Rite Rezchecker [30]) under external uniform illumination. The captured color patch values were compared to known values and the color error, or difference between measured and reference values, was calculated in the CIELAB color space. This framework was chosen as it is a device-independent technique to characterize color reproduction accuracy of each system. CIELAB uses ***L*** for lightness (or luminosity in the z axis), and x- and y-axis are cube root transforms of color data in a* and b*, color-opponent dimensions. Generally, the perceptible color difference is approximately equal to the geometric or Euclidean distance between reference and measured L*a*b* values, the two metrics here $\Delta E{*}_{ab}=[\sqrt{\left({\left(\Delta L*\right)}^{2}+{\left(\Delta a*\right)}^{2}+{\left(\Delta b*\right)}^{2}\right)}]$, accounts for luminance difference and $\Delta C{*}_{ab}=[\sqrt{\left({\left(\Delta a*\right)}^{2}+{\left(\Delta b*\right)}^{2}\right)}]$ does not account for luminance [31].

An optical sliding rail platform constructed from commercial off the shelf components allowed for precise 3-axis control of the imaging target and imaging systems being evaluated. Working distance was measured with a Bosch DLR130 Laser Distance Measurer, which has a range of 50 to 39,624 mm and accuracy of ±1.6 mm. Standard imaging targets previously described were mounted on thin magnetic sheeting for efficient mounting and removal. An off-the-shelf illumination system was adapted using White (D50/5000K) LED sources, a pair of modified portable work lights with custom 3D printed optical table mounts, and 100 x 100 mm 120 grit ground glass diffusers were used to improve light uniformity. These light sources were positioned between 20° and 40° from the midline plane of imager to provide uniform lighting on the imaging targets. A BK Precisions 615 light meter, which has a flat detector surface, was used to capture incident light (lux) on the imaging targets for use in the image quality calculations. Objective image quality parameters were calculated with these targets using the Imatest 3.10 and Matlab 2013b software packages [29].

### Characterization and Quantification of Illumination Source

The spectra of each system was characterized using an Ocean Optics USB4000 spectrometer coupled to a 1.0mm diameter fiber mounted concentric with the illumination beam. Five sets of spectral measurements with dark correction were captured for each system, with an integration time of 100 ms. In order to prevent oversaturation, varying neutral density filters were placed in the optical pathway to attenuate the light signal.

Uniformity of the beam was assessed by imaging the beam from the respective light source onto a diffuser plate set at the working distance of each system. Using an optical rail system, we were able to precisely control the working distances and alignment of our testing platform. A 12 MP color CMOS detector was placed concentric with the center of the illumination beam, and a diffuser was placed in the beam path between the CMOS detector and the light source being tested. The CMOS detector was placed at a distance that allowed the detector system’s lens to be at minimal zoom, which maximized the field of view. Two images of the beam were captured: one with the diffuser only to characterize the beam shape, and a second with a centimeter grid placed over the diffuser to allow for beam width determination. Using the Uniformity function in Imatest 3.10, we then assessed the full width half maximum (FWHM) beam diameter and were able to generate contour maps of deviations from the central Color Temperature in the illumination pattern [29].

### Safety

The POCkeT Colposcope and other digital colposcopy systems use broadband white light sources, so there is little potential for eye and tissue damage. Maximum permissible emissions (MPE) were measured and calculated using ANSI/IESNA RP-27.3–07 Recommended Practice for Photobiological Safety for Lamps guidelines [32]. Each system produced wavelengths in the visible and NIR, with the 2 primary safety concerns being Blue Light Corneal Hazard, *B*(*λ*) and Retinal Thermal Hazard, R(*λ*). The Optical Power (mW) and Irradiance (mW/cm^2^) of each system were captured with a Coherent Fieldmax II-TO meter (Coherent Inc., Santa Clara CA) and OP-VIS2 and OP-IR2 detectors. The photochemical blue light hazard metric is spectrally weighted for radiance, $L_{B}=\sum_{300nm}^{700nm}L_{\lambda }*B(\lambda )*\Delta \lambda$. *L*_*B*_ is weighted radiance in W*cm^2^sr, *L*_*λ*_ is the spectral radiance with units W*cm^-2^*nm^-1^, Δ*λ* is the calculation interval in nm, and *B*(*λ*), is a weighted function of blue light hazard from 300 nm to 700 nm [32]. The retinal thermal hazard metric is spectrally weighted for irradiance, $L_{R}=\sum_{400nm}^{1400nm}L_{\lambda }*R(\lambda )*\Delta \lambda$. *L*_*R*_ is the weighted irradiance in W*cm^2^, *L*_*λ*_ is the spectral radiance in W*cm^-2^*nm^-1^, Δ*λ* is the calculation interval in nm, and R(*λ*),is a weighted function of burn hazard from 400 to 1400 nm [32]. The risk group for exemption is 10 W/cm^2^sr for Blue Light Hazard L_B_ at 10,000 second exposure timeframe and for Retinal Thermal *L*_*R*_ is 2.8/*α* W/cm^2^sr or 25.45 W/cm^2^sr for a 10 second exposure timeframe [32]. Here *α* is the angular subtense of source in radian defined by 50% peak radiance points. L as the beam diameter and the working distance R, and the subtended angle *α* = L / R (radians). However, from ANSI/IESNA RP-27.3–07 guidelines if the angle *α* > 0.11 radians, 0.11 radian is used and if *α* < 0.011 radian, 0.011 is used in the calculation for the MPE limits [32]. The results from the MPE calculations would provide the optical safety of each system’s illumination source and if beyond the “Exempt” limit need to have precautions taken to prevent patient and operator eye damage including the appropriate PPE (personal protective equipment) and device labeling.

### In-vitro and pilot in vivo concordant imaging of the cervix

In-vitro imaging of simulated high grade and normal cervices in a life scale educational mannequin (Gaumard Zoe S504.100 (Gaumard Scientific, Miami FL)) was performed using the POCkeT Colposcope’s system and other digital colposcopy and cervicography systems. Images captured from this testing help provide confirmation that the POCkeT Colposcope’s could function in enclosed space at the designed working distance and compatibility with a speculum. Specular reflection was quantified as the percentage of “hot pixels” (with luminous channel intensity > 250) at 11 even steps of optical power intensities for the POCkeT Colposcope’s white and green field illumination of in-vitro simulated cervix at a working distance of 40 mm. The percentage of specular reflection was plotted as a function of optical power and using the empirical derived limit of 1% specular reflection from the commercial high-end Leisegang Optik 2 digital colposcope system was used as the benchmark.

A pilot exploratory study in human subjects was conducted to demonstrate feasibility of the POCkeT Colposcope to capture comparable images to a high-end digital colposcope under an institutional IRB approved protocol, consent process, and data storage system (Pro00008173) at the Duke University Medical Center (DUMC). Adult subjects (18–65 years of age) undergoing routine colposcopy and/or LEEP treatment provided written informed consent using an institutional IRB approved consent form for concurrent imaging with the POCkeT Colposcope and clinic’s high end digital colposcope system (Leisegang Optik 2). Participating patients are also provided a copy of their signed consent form. The original consent form is kept in a secured research binder that also is also used to document the consent process and procedure followed the approved protocol in a locked cabinet residing in a locked office that only IRB approved on the study have access to. All data was de-identified of all Protected Health Information (PHI) and a new study randomized study ID is generated before being stored on an IRB approved encrypted and password protected server. As part of standard of care, a speculum was placed and any blood or mucous was removed from the cervix using a fox swab. A 5% acetic acid solution was then applied followed by digital image capture with the Leisegang Optik 2 at 3.75X magnification under white and green light illumination. The high-end colposcope was moved out of position, and the POCkeT Colposcope set at 4-6X magnification was placed into the speculum to capture white and green field images. This process added between 1–2 minutes to the overall procedure time. The procedure continued with biopsy sample collection and/or LEEP treatment as part of the standard protocol. Cervix specimens were processed, immuno-stained for Ki-67(a nuclear protein marker for cellular proliferation) and p16 (a cyclin-dependent kinase-4 inhibitor that is a surrogate marker for the presence of HPV infection), and read by institutional pathologists as the gold-standard diagnostic reference [33].

### Image Processing

Color corrections are performed on the POCkeT Colposcope images using Adobe Photoshop CS6’s “color match” function using the Leisegang Optik 2’s captured image as the color reference. First, the reference image captured by the Leisegang Optik 2 is opened in Adobe Photoshop CS6, and the circular outline of the cervix is traced using the “lasso” tool with a surface area of approximately 40% of the field of view selected. The concordant 5.0MP POCkeT Colposcope image is then opened, a work space layer is created and overlaid on the image. In this layer the “lasso” tool is used to trace the circular outline of the cervix with a surface area of approximately 60% of the field of view selected. Once these regions of interests are selected, the “match color” tool is selected from the “Image” tab and subsequent toolset “Adjustments”. The “Source” image is the ROI selected from the Leisegang Optik 2 and the “Layer” image is the ROI selected to be color corrected from the 5.0MP POCkeT Colposcope image. The luminance, color intensity, and fade are left at default values of 100, 100, and 0, respectively. The “layer” mode is changed from “normal” to “luminosity” and intensity levels are adjusted from 20 to 240 (from base of 0 to 254). These settings were optimized using a NIST calibrated X-Rite Rezchecker color calibration target to minimize the L*a*b color space errors, ΔE*_ab_ and ΔC*_ab_.

## Results

**Fig 2:**
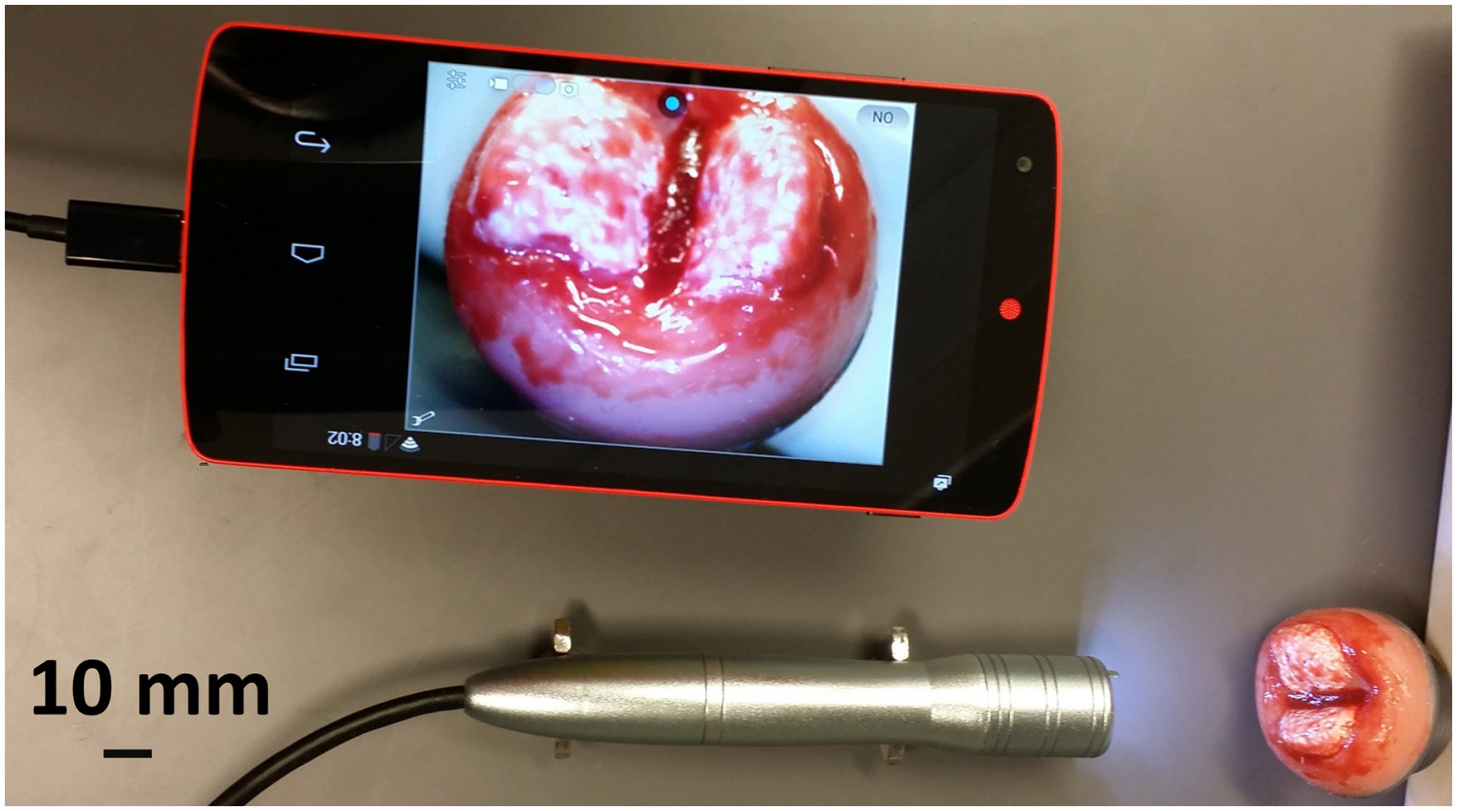
A comparison of different colposcopy platforms across resolution, diagonal field of view, and working distance with a representative image of a USAF1951 test target from each. Representative images of USAF21951 resolution target with the Leisegang Optik 2 at 3.75X magnification (A), 5.0MP POCkeT Colposcope at 4X (B), 2.0MP POCkeT Colposcope at 4X (C), Apple iPhone 5S at 3.5X (D), and Canon SX50HS at 2.5X (E). Our POCkeT Colposcope system (2.0MP as blue circles and 5.0MP as purple circles) have the capability to resolve features as small as 10 micron (F) comparable to the highest magnification of clinical systems. Diagonal field of view (FOV) of Leisegang Optik 2 (G) (Red Diamond) has the largest of any of the digital systems, because of the large image sensor format and 3:2 aspect ratio and would produce images 17% wider than the comparable system with 4:3 aspect ratio. Our POCkeT Colposcope system is still capable of providing a FOV of at least 30 mm, comparable resolving power for identification of potential lesions, while at much closer working distances (H) between 50 to 10 mm. Images have been cropped to better demonstration magnification of ROI (region of interests).

Baseline imaging characteristics of the digital colposcope (Leisegang Optik 2), the analog colposcope (Wallach Zoomscope), the digital cervicography devices (Canon SX50HS and Apple iPhone 5S), and the 2.0MP and 5.0MP POCkeT Colposcope (PC) are shown in Fig 2. Representative images of the USAF 1951 target are shown in Fig 2A–2E. Fig 2F shows the system resolution as a function of magnification. As our reference, the Leisegang Optik 2 at 7.5X magnification can resolve features as fine as 22.1 micron, the digital cervicography systems are not able to resolve as fine of features with resolvable objects at 34.97 to 44.25 micron for the iPhone 5S and Canon SX50HS, respectively. At a working distance of 30 mm, both POCkeT Colposcope generations were able to resolve features close to the gold standard digital colposcope with features sized 27.86 and 24.8 micron thickness, for 2.0MP and 5.0MP POCkeT Colposcope systems respectively. The commercial colposcope systems have generally the longest working distances of ~300 mm throughout their range of magnifications and the POCkeT Colposcope has the shortest working distance of 10–50 mm (Fig 2H). Furthermore, our POCkeT Colposcope system can be moved closer to any suspicious lesions on the cervix to allow for even higher magnification at working distances of 10 mm.

The 5.0MP POCkeT Colposcope has comparable and minimal barrel distortion of 1.14% when compared to the Leisegang Optik 2 of ~ 0.5% (Table 2) and that features found at the peripheral of the cervical image are not overtly distorted to prevent proper colposcopic interpretation of lesion margins, shape, and vascular patterns. These are key geometric features often used to stage cervical pre-cancers.

**Table 2:** Comparison of the Radial Lens Distortion between colposcopy systems.

System	Lens Distortion (%, Type)
Leisegang Optik 2	-0.601, Barrel
Canon SX50HS	-0.796, Barrel
Apple iPhone 5S	-0.935, Barrel
2.0MP POCkeT Colposcope	-4.49, Barrel
5.0MP POCkeT Colposcope	-1.14, Barrel

When compared with the gold standard Leisegang Optik 2 digital colposcope; all other systems had small amount of barrel distortion of less than 5% and the 2.0MP POCkeT Colposcope had the highest barrel distortion.

**Fig 3:**
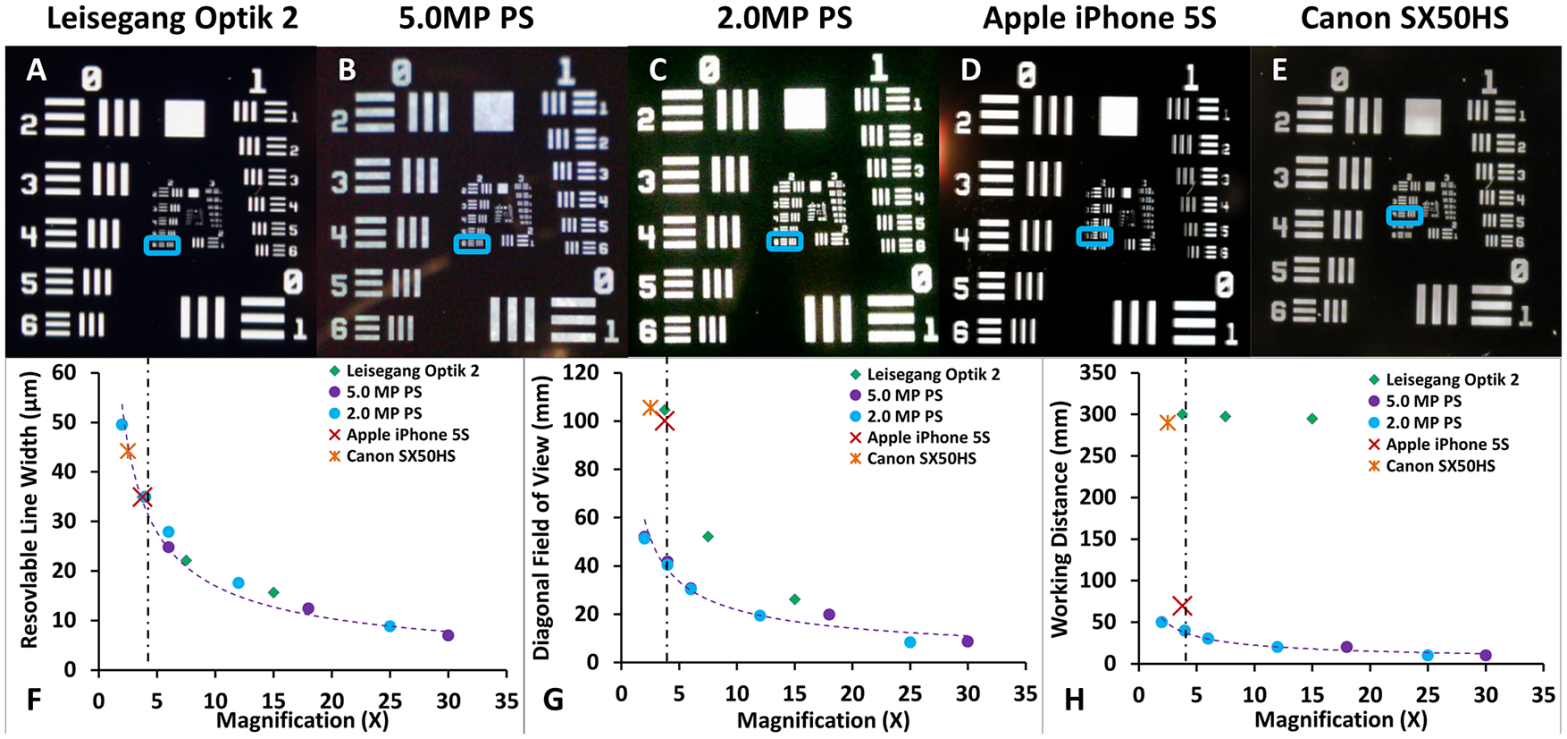
Color accuracy was assessed using NIST calibrated color target (X-Rite Rezchecker) for all digital colposcopy systems. The X-Rite Rezchecker color target was imaged under D50 or White 5000K illumination, (A) Leisegang Optik 2, (B) 5.0MP POCkeT Colposcope (C) 2.0MP POCkeT Colposcope, (D) Canon SX50HS, and (E) Apple iPhone 5S. Perceptible color difference is therefore the approximately equal to the Euclidean distance between reference and measured L*a*b* values. Δ*C*_*ab*_ (F) green bars are for mean and purple bars for max color difference, without accounting for any luminance differences present and ΔE*_ab_ (G) blue bars are for mean and red bars for max color differnece, with weighting for any luminance differences. The best performing is the Leisegang, with mean perceptible color difference Δ*C*_*ab*_ of 2.9 (F) and Δ*E*_*ab*_ of 5.24 (G), accounting for luminance. Generally, when luminance was included, the error increased 2.5 times.

The opacity and color of potential lesions are important characteristics often used to stage cervical pre-cancers and accurate color reproduction is needed to prevent misclassification by the clinician. Accuracy in white balance is important as low-grade lesions are often characterized as having snow to bright white coloration when compared to high-grade lesions that are oyster gray in color [26, 34, 35]. In white field imaging, red color reproduction and in green field imaging green color reproduction is important for detection underlying vasculature [34]. Representative images of the X-Rite Rezchecker Matte Color Target can be found in Fig 3A–3E. Note the marked difference in color of the 2.0MP POCkeT Colposcope as compared to the other systems. The color reproduction was nearly 3 times worse than the reference Leisegang Optik 2 in both ΔC_ab_ (color error without accounting for difference in luminance between the reference image and the test image) and ΔE_ab_ (color error accounting for difference in luminance between the reference image and the test image) (Fig 3F and 3G). The 5.0MP POCkeT Colposcope had only 1.5 times higher color error for both parameters when compared to the Leisegang Optik 2 reference system. In terms of green and red color accuracy important for vasculature identification, the Leisegang Optik 2 performed the best with the least error and additionally had the least baseline white balance error (smallest ΔC_ab_ error in the white to gray patches in the center of the target) and the 2.0MP POCkeT Colposcope having the worst white balance error. The 5.0MP POCkeT Colposcope had the second best white balance error and performed in the middle of the pack in terms of green and red color accuracy, however this system would benefit from some white balance, green, and red color algorithm adjustments to better match the high-end system. Interestingly, all 3 systems had the highest color reproduction error in the blue and purple color ranges.

**Fig 4:**
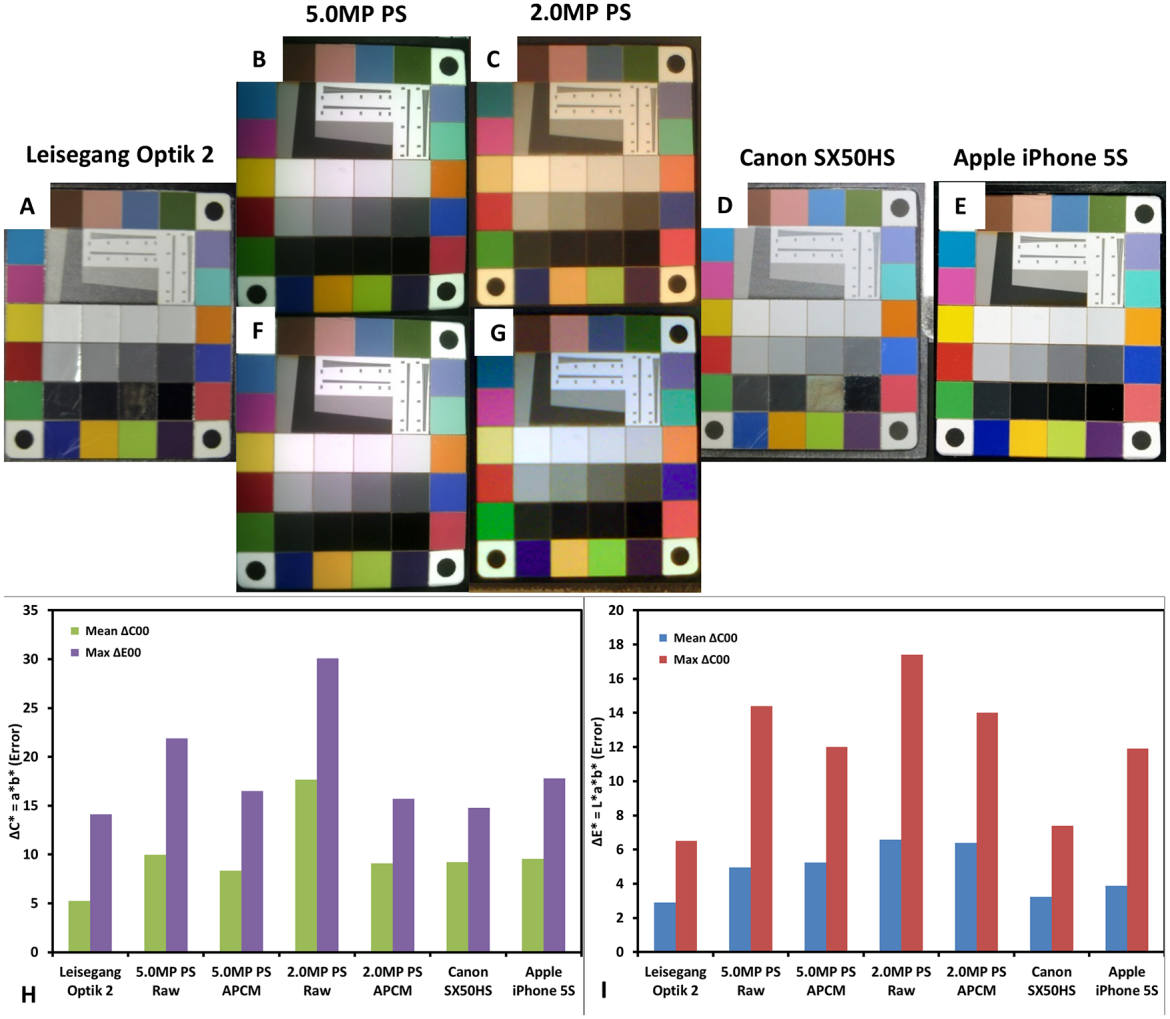
Performance of a color matching algorithm for use on the POCkeT Colposcope images to the high-end Leisegang Optik 2. The X-Rite Rezchecker target with NIST calibrated color patches captured in jpeg format by both Leisegang Optik 2 and our 5.0MP TVDC system with on-board white balance and color correction algorithm’s enabled. The pairs are the color matched using Adobe Photoshop CS6, 5.0MP POCkeT Colposcope images on the middle (BC), and 2.0MP POCkeT Colposcope images on the right (DE). In panel FG, we recalculated the Δ*C*_*ab*_ (F) green bars for mean and purple bars for max color difference, the darker color bars are the post-color match values, without accounting for any luminance differences present. ΔE*_ab_ (G) blue bars for mean and red bars for max color differnece, again darker color bars are post-CCM,with weighting for any luminance differences. Note, that post color match values were generally lower for both metric values and closer to the Leisegang Optik 2 reference values.

Due to the fact that most if not all the digital colposcopy and/or cervicography systems have integrated onboard white balance and color correction algorithms we set out to try and correct for these differences by calibrating to a NIST color target and Imatest 3.10. We unfortunately do not have access or currently the ability to get the raw format images and have to do post-processing work with the images. In the pre- and post-color corrected image shown below (Fig 4), Adobe Photoshop CS6’s “color match” function was used to correct coloration of concordant image pairs using the Leisegang Optik 2 captured images set as the reference.

**Fig 5:**
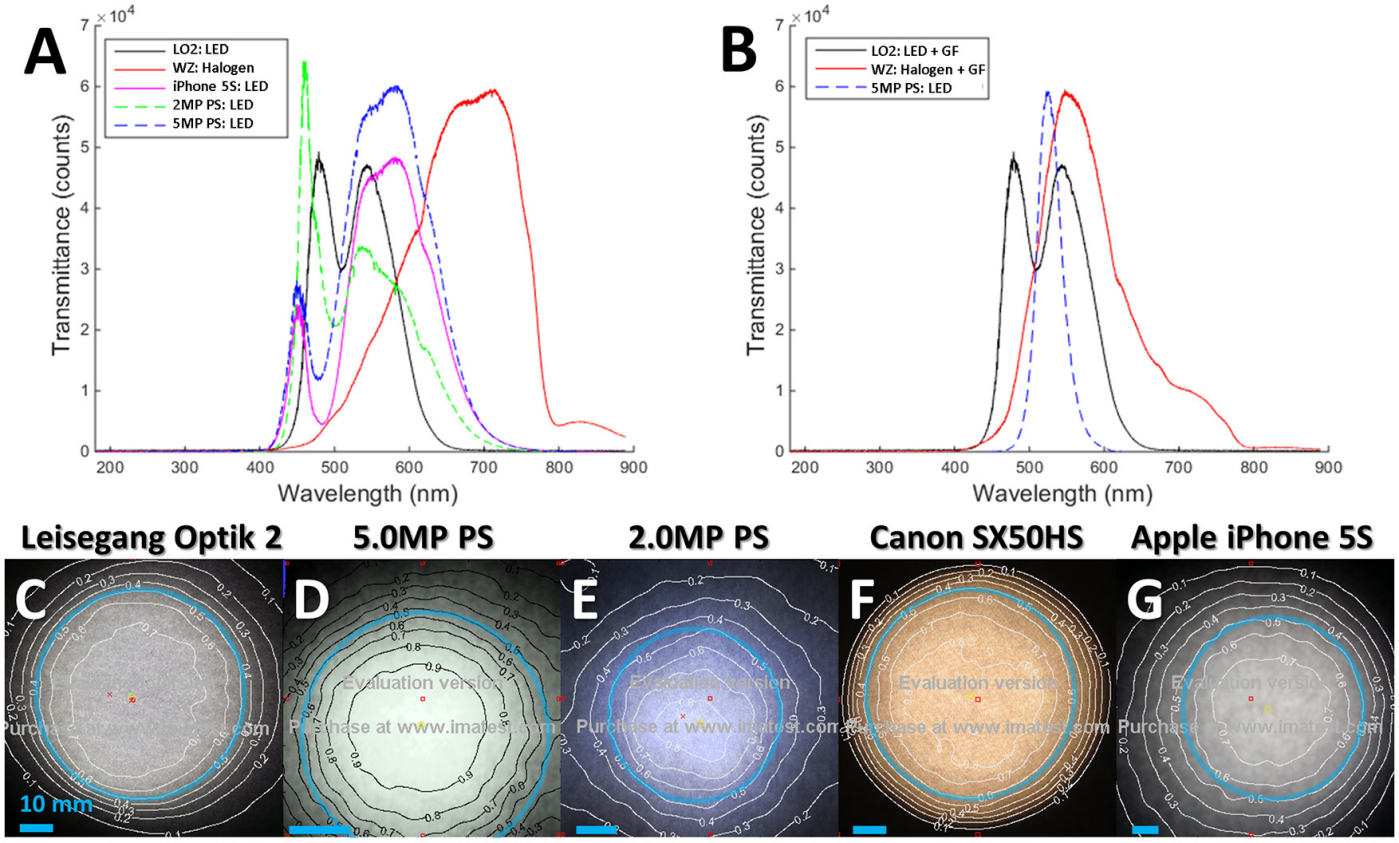
Representative spectra and beam characteristics from white and green field illumination beam characteristics of compared digital colposcopy systems. (A) Examination of the white spectra of the various digital colposcopy systems: Leisegang Optik 2 (black) with a 5000K white light emitting diode (LED), Wallach Zoomscope halogen source (red), Apple iPhone 5S white LED source (purple), 2.0MP POCkeT Colposcope White LED with 5700K Color Temperature (green), and 5.0MP POCkeT Colposcope with white 5000K LED (blue). (B) Examination of the green field illumination spectra from the Leisegang Optik 2 (LO2) clinical digital colposcope, which has a 5000K White LED source in solid black, the Wallace colposcope using a halogen source (red line), both using broad bass-pass filters. Our 5.0MP POCkeT Colposcope with dedicated green LEDs (dashed blue). (C to G) Luminance contour plots normalized from 0 to 1, the Full Width Half Maximum (FWHM) beam diameter is defined as 50% contour luminance for each illumination systems at their respective working distances. The light blue rings indicate the location of the FWHM and scale bars = 10 mm. The Leisegang Optik 2 (C) had a FWHM beam diameter of 62 mm at Working Distance (WD) of 300 mm, 5.0 MP POCkeT Colposcope (D) had a FHWM beam diameter of 40.2 mm at WD of 40 mm, 2.0MP POCkeT Colposcope (E) had a FWHM beam diameter of 42.8 mm at WD of 40 mm, Wallach Tristar (F) a FWHM beam diameter of 64.2 mm at WD of 300 mm and the Apple iPhone 5S (G) a FWHM beam diameter of 70.2 mm at WD of 70 mm.

Examination of the illumination strategies revealed a distinct difference in the spectra of the Wallach Zoomscope (halogen source) when compared to all other systems which used white LEDs (Fig 5A). The illumination spectra for green field was markedly different for the 3 systems with that capability (Fig 5B). The POCkeT Colposcope delivers a narrow band center around green 530±30nm (central λ± FWHM), the Leisegang broader band-pass with spectra in blue to green 550±100 nm and Wallach colposcopes use a long-pass strategy with mostly green and red at > 500 nm. Selective illumination of the cervix with green light leverages increased absorption by hemoglobin in that spectra when compared to other bands of visible light and leading to enhanced contrast of vasculature (appearing dark against a light background) [36]. The use of a narrower band of illumination helps enhances the vasculature contrast when compared to broader illumination [37]. Non-uniform light distribution is a major hurdle in image processing for lesion margin and vasculature extraction from digital cervical images [26, 38, 39]. Thus, beam characterization of each systems illumination strategy provide was examined in the case of white field illumination with contour plots of luminance overlaid on images on a diffuse target (Fig 5C to 5G). The FWHM corresponds to the 0.5 contour line (50% luminance). Both commercial clinical colposcopes use collimating lenses to focus the light and are able to produce tighter beam spots. The order of a magnitude shorter working distance of the POCkeT Colposcope systems compared to the commercial colposcopes enables to the omission of a collimating lens.

The Wallach Tristar produced the highest optical power and irradiance measure in our testing, which is required due to the long working distance (300–330 mm) and halogen source see Table 3. Similarly, the Leisegang Optik 2 produces the second highest optical power and irradiance, but the light source is a more efficient LED source and at working distance of 300 mm from the cervix. The 5.0MP POCkeT Colposcope does not need to produce similar optical radiance and irradiance levels to adequately illuminate the cervix due to the shorter working distance, requires less power, and minimizes any potential tissue heating with the use of smaller efficient LEDs.

**Table 3:** Comparison of consumed electrical power, optical power, irradiance, maximum illumination, and beam diameter across the digital colposcope systems.

System	Illumination type	Electrical Power (W)	Optical Power (mW)	Irradiance (mW/cm^2^)	Maximum Illumination (ft-candle)	Beam Diameter (FWHM) (mm)
Leisegang Optik 2	White LED 5000k	18	79.3	183.9	2060	62
	LP Green Filter	18	32.1	77.3	1112	62
Wallach Tristar	Halogen	150	129.9	265.5	1683	64.2
	LP Green Filter	150	19.9	40.8	613	64.2
Canon SX50HS	Halogen*	Same 150 W Halogen Source as Wallach Zoomscope				
Apple iPhone 5S Flash	White LED/Xenon	N/A	13.2	24.1	147	70
2.0MP POCkeT Colposcope	White LED 5700k	1	1.22	2.45	445	42.8
5.0MP POCkeT Colposcope	White LED 5000k	2	10.7	23.6	749	40.2
	Green LEDs	1	3.61	9.45	467	40.2

Our 5.0MP POCkeT Colposcope has the flexibility to produce nearly comparable optical power (mW), irradiance (mW/cm^2^), and maximum illumination (ft-candle) to the commercial colposcope systems for both white and green field illumination and could produce a sufficient size FWHM beam spot of 40.2 mm to illuminate the whole cervix, generally 30–35 mm in diameter.

* For digital cervicography with the Canon SX50HS at Kilimanjaro Christian Medical Center, the 150 W halogen source from the analog Wallach Tristar colposcope was used for illumination

Maximum Permissible Emissions from all systems were characterized and the ***L***_***B***_ blue light hazard for all systems fell into the Exempt Category and less than the 10 W/cm^2^sr limit. The Wallach Zoomscope, however, with a halogen source, superseded the ***L***_***R***_ Retinal Thermal Hazard limit for Exempt Category of 25.45 W/cm^2^sr by a factor of 4 (Table 4). Our POCkeT Colposcope systems were significantly below the danger threshold for both blue and red light hazards and do not need eye safety labeling while providing sufficient illumination of the cervix at lower electrical power consumption than commercial systems.

**Table 4:** Comparison of Light Source Safety Maximum Permissible Emissions testing for the digital colposcopy systems.

System	L_B_ Blue Light Hazard (mW/cm^2^sr)	L_R_ Retinal Thermal hazard (W/cm^2^sr)
Leisegang Optik 2	239.7	24.0
5.0MP POCkeT Colposcope	4.61	0.52
2.0MP POCkeT Colposcope	4.78	0.48
Canon SX50HS*	960.0*	150.7*
Apple iPhone 5S Flash	36.3	3.68

The limits for the exempt classification at subtended angle of 0.11 radians for L_B_ at 10 W/cm^2^sr and L_R_ 25.45 W/cm^2^sr.

* For digital cervicography with the Canon SX50HS at KCMC, the 150 W halogen source was used for illumination from the analog colposcope, the Wallach Tristar.

**Fig 6:**
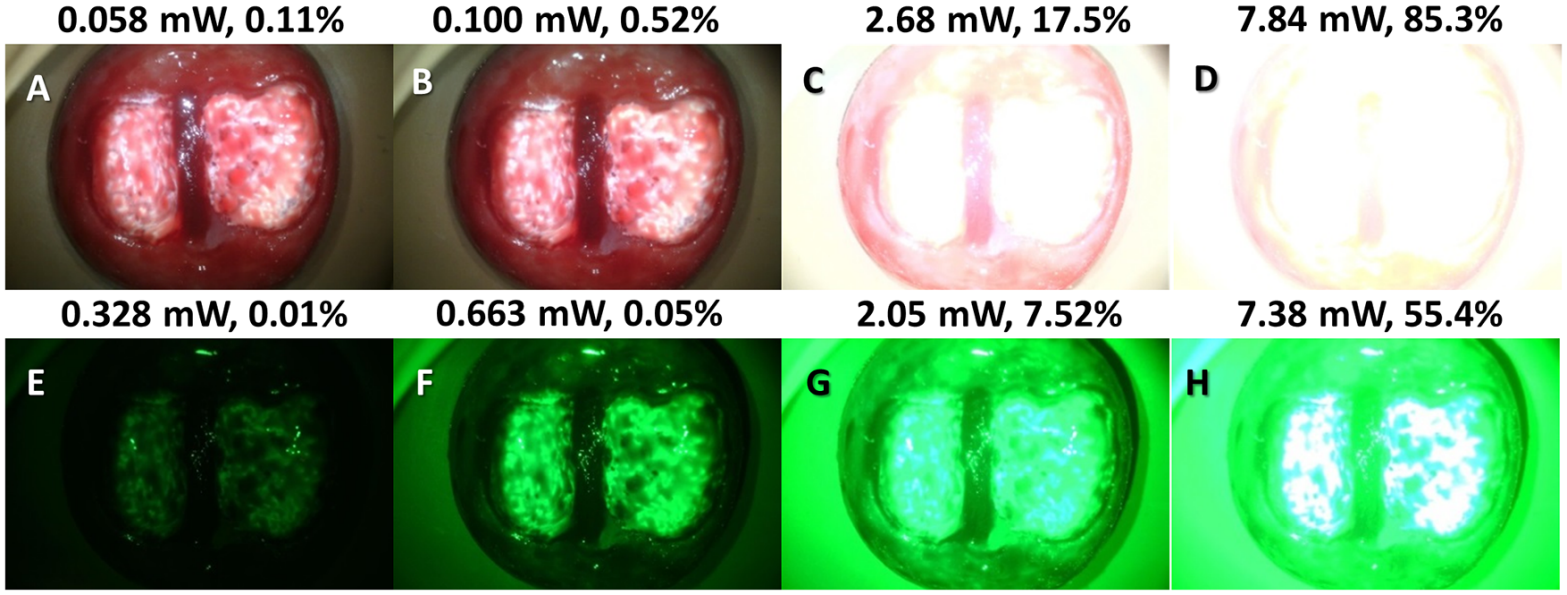
Representative images from the percentage of specular reflection as a function of optical power. The top most panels (ABCD) correspond to White 5000K color temperature LED illumination and bottom panels (EFGH) corresponding to Green LED illumination with the POCkeT Colposcope on a simulated HSIL/CIN2+ cervix. From left to the right is increasing optical power achieved with PWM (pulse width modulation) control and shows the expected increase in specular reflection to above the threshold of failure of 0.5% for white light and 0.05% for green light, set by the commericial system.

We also quantified the specular reflection produced by our system as a function of optical power for both White and Green LED illumination by taking a series of test images on a simulated HSIL/CIN2+ cervix. Specular reflection was defined as the percentage of “hot pixels” at intensity of 250 or higher divided by all pixels at a given optical power density. Based on the empirical evaluation of the commercial system, a target threshold of specular reflection of 0.5% for white light and 0.05% for green light were the goals for our system for comparable performance. The optical power limit with our cross-polarized strategy for White LED illumination was determined to be 0.100 mW and for Green LED illumination was 0.663mW, respectively (Fig 6).

**Fig 7:**
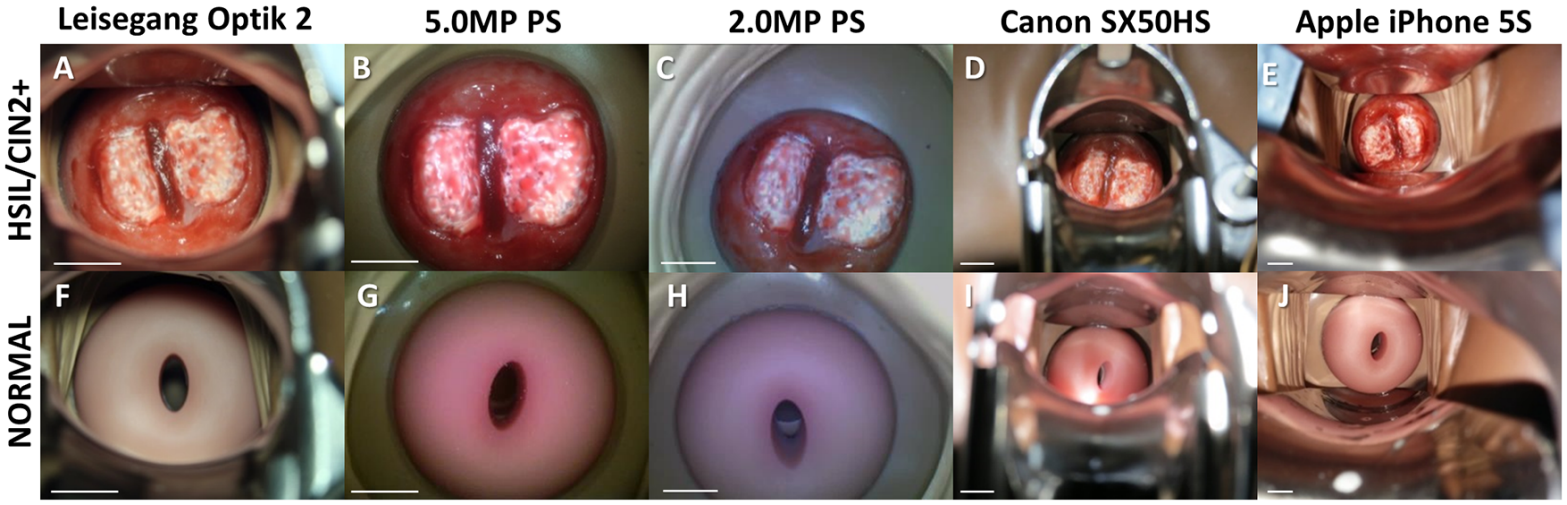
Representative cervical images captured the digital colposcopy systems on simulated normal and high grade simulated cervices. (A-E) Simulated HSIL Cervix in Gaumard Zoe Gynecologic Simulator, (F-J) Simulated Normal cervix in Gaumard Zoe Gynecologic Simulator. Paired images from a Leisegang Optik 2 Digital Colposcope (AF) at 3.75X at a working distance of 300 mm, our 5.0MP POCkeT Colposcope (BG) at 4X at working distance of 30 mm, our 2.0MP POCkeT Colposcope (CH) at 4X at working distance of 30 mm, a Canon SX50HS digital camera (DI) at 2.75X at a working distance of 300 mm, and an Apple iPhone 5S (EJ) at 3.75X at a working distance of 70 mm. There was no color correction performed on the POCkeT Colposcope images. The target illumination intensity was set at the 1% specular reflection threshold for all systems.

Viability of cervical imaging POCkeT Colposcope prototypes against current clinical imaging systems was performed in a life-size Gaumard Zoe Gynecologic Simulator Mannequin with high grade squamous intraepithelial lesion (HSIL) lesions (Fig 7A–7E) and normal cervix (Fig 7F–7J). The Canon SX50HS and Apple iPhone 5S are not able to fill the detector frame due to limitations with their respective optical systems. The POCkeT Colposcope systems (Fig 7B, 7G, 7C and 7H) have the ability to magnify the cervix to fill the imaging frame and can adjust to provide higher magnification of suspicious sites like the high-end commercial system and with comparable image quality to the commercial clinical colposcopes.

**Fig 8:**
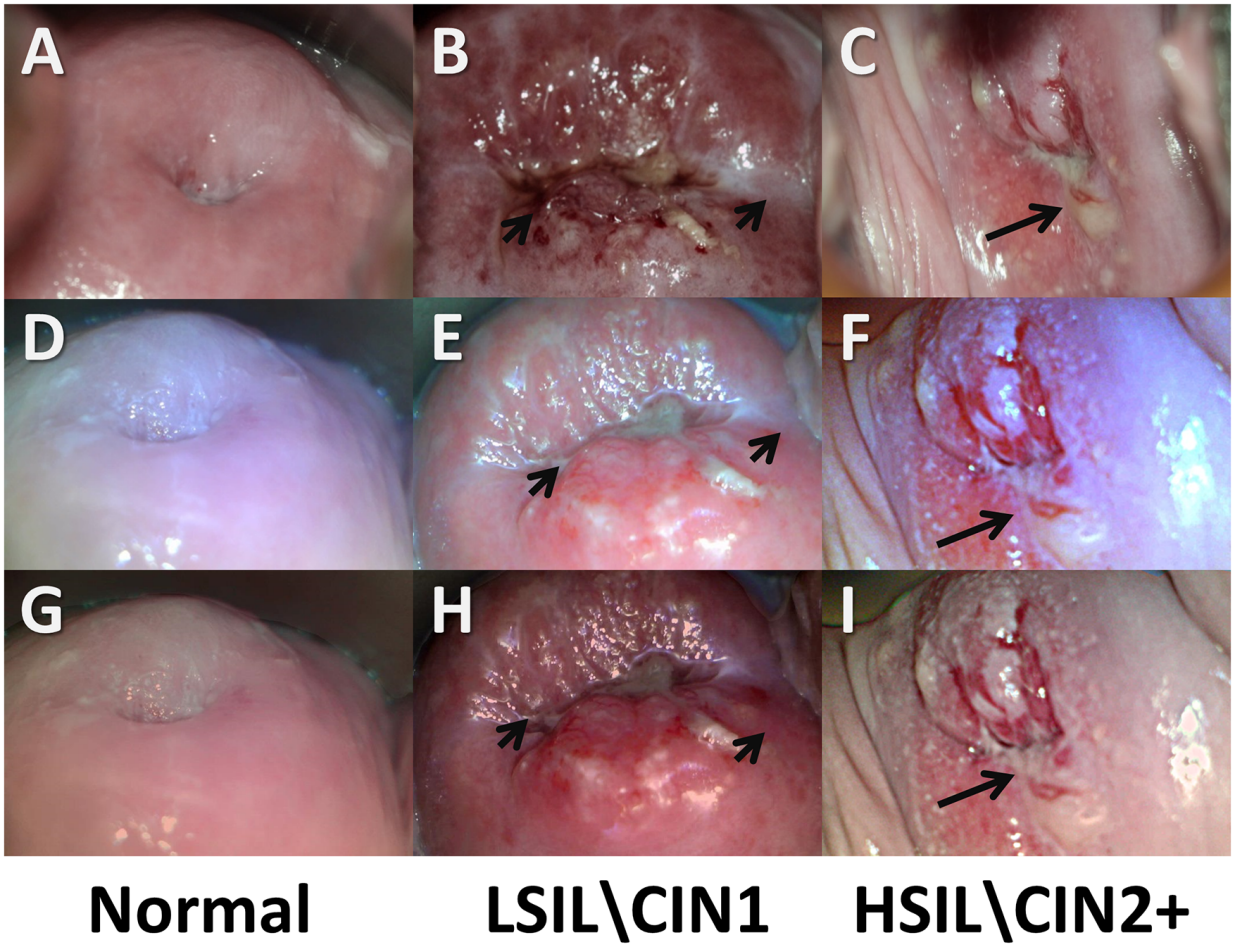
Representative cervical images captured with the high-end digital colposcope at 3.75X magnification and our 2.0MP POCkeT Colposcope. (ABC,GHI) Representative cervical images captured at 3.75 X with Leisegang Optik 2 Digital Colposcope at a working distance of 300 mm and (DEF, JKL) concordant images at 4X with 2.0MP POCkeT Colposcope at a working distance of 40 mm. Normal Cervix (ADGJ), biopsy confirmed LSIL/CIN 1 (BEHK) at 3 o’clock position and normal but suspicious lesion at 7 o’clock, and HSIL/CIN3 confirmed LEEP pathology (CFIL) at 4 to 5 o’clock position. Color correction was performed on the POCkeT Colposcope’s images using color match function in Adobe Photoshop, GHI, JKL and original images in ABC,DEF. The white light illumination power for the POCkeT Colposcope are set at a 1.0% specular reflection threshold.

**Fig 9:**
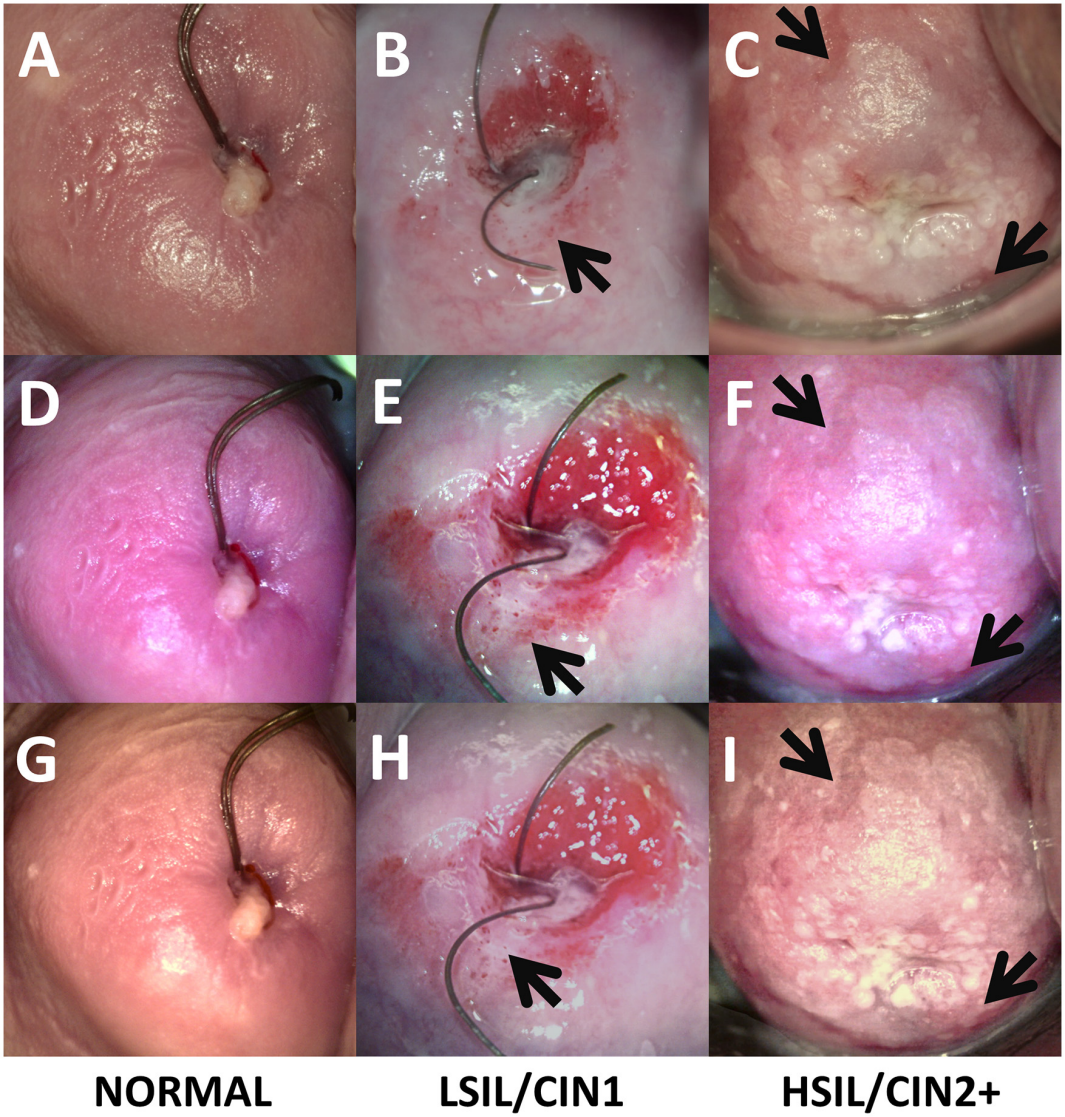
Representative cervical images captured with the high-end digital colposcope at 3.75X magnification and our 5.0MP POCkeT Colposcope. (ABC,GHI) Representative cervical images captured at 3.75 X with Leisegang Optik 2 Digital Colposcope at a working distance of 300 mm and (DEF, JKL) concordant images at 4X with 5.0MP POCkeT Colposcope at a working distance of 40 mm. Normal Cervix (ADGJ), biopsy confirmed LSIL/CIN 1 (BEHK) at 1 o’clock position, and biopsy confirmed HSIL/CIN 3 pathology (CFIL) confirmed at the 8 and 11 o’clock. Color correction was performed on the POCkeT Colposcopes images using color match function in Adobe Photoshop, GHI, JKL and original images in ABC, DEF. The white light illumination power for the POCkeT Colposcope was set at the level for 1.0% specular reflection threshold, however we have moved to the 0.5% specular threshold for all subsequent cases.

Representative concordant cervical images from our 2.0MP (Fig 8D, 8E, 8F, 8J, 8K and 8L) and 5.0MP (Fig 9D, 9E, 9F, 9J, 9K and 9L) POCkeT Colposcope systems were compared to the matched image from the Leisegang Optik 2 18.0MP system (Figs 8A, 8B, 8C, 8G, 8H, 8I and 9A, 9B, 9C, 9G, 9H, 9I) with pathology confirmed lesion classification of Normal (column AEGJ), low grade squamous intraepithelial lesion\ cervical intraepithelial neoplasia (LSIL\CIN1) (Figs 8B, 8E, 8H, 8K and 9B, 9E, 9H, 9K), and high grade squamous intraepithelial lesion\ cervical intraepithelial neoplasia 2+ (HSIL\CIN2+) (Figs 8C, 8F, 8I, 8L and 9C, 9F, 9I, 9L) for each image set comparison. Original images are ABC, DEF and post color corrected matrix in rows GHI, JKL for both the 2.0 and 5.0MP POCkeT Colposcopes. Black arrows indicate suspicious regions due to heterogeneity in acetowhitening and/or vascular abnormality.

**Fig 10:**
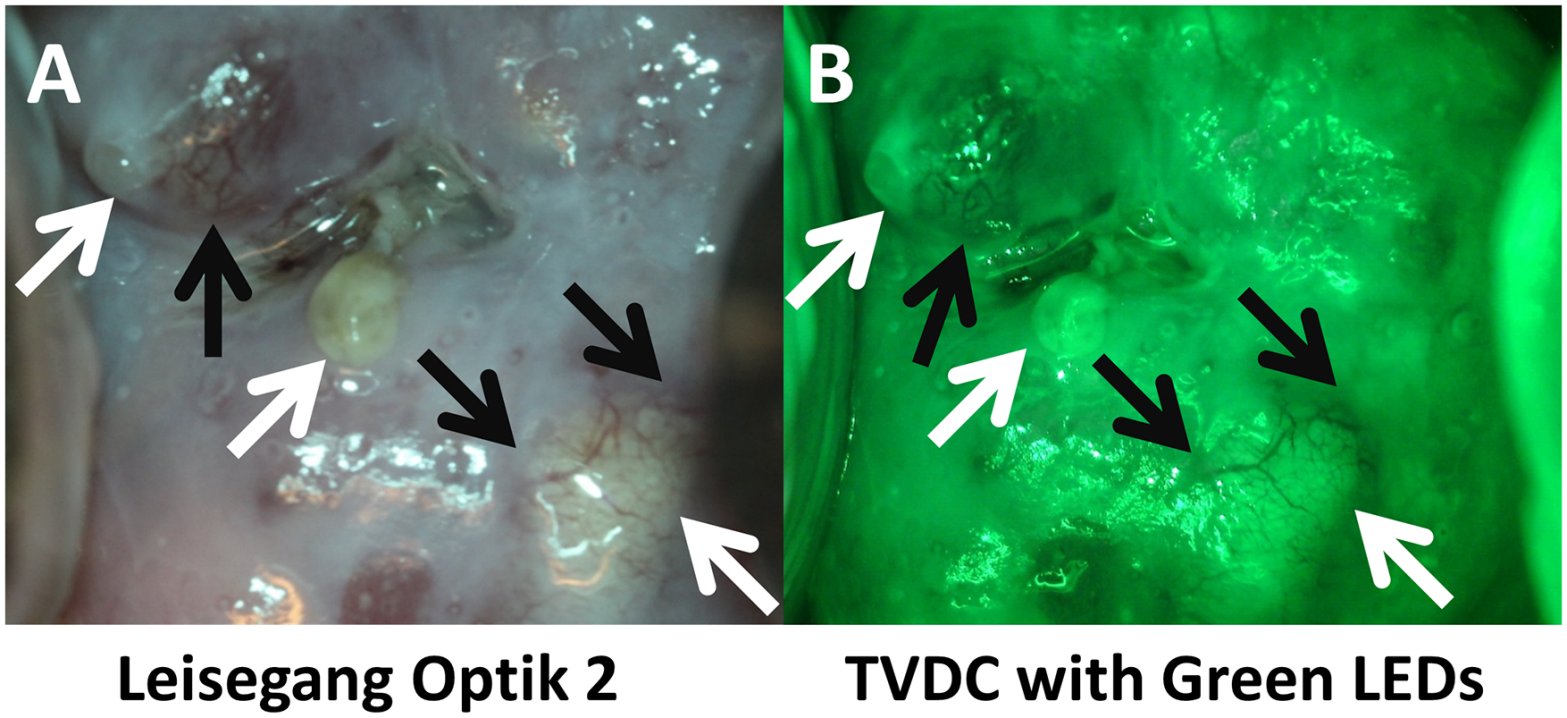
Comparison of green field images of a Nabothian cyst between the high-end digital colposcope and our POCkeT Colposcope. The following image panel is of a biopsy confirmed normal cervix with Nabothian cyst (yellow and clear nodules) taken by the Leisegang Optik 2(AB) at 3.75X magnification and the 5.0MP POCkeT Colposcopes (CD) at 4X magnification. Black arrows indicate prominent vascular features and note the enhanced contrast with the “green field” illumination strategies by both systems. Both systems use White 5000K color temperature LEDs, while a green LED is use by the 5.0MP POCkeT Colposcope when compared to the short-pass Red filter used by the Leisegang Optik 2. Color correction was performed on the POCkeT Colposcope images using the color match function in Adobe Photoshop, GHI, JKL and original images in ABC,DEF. Here the illumination power threshold were set at 1% and 0.5% specular reflection level, respectively for white and green field illumination with the POCkeT Colposcope. We have now moved to a 0.5% and 0.05% specular reflection threshold for illumination power for all subsequent clinical cases.

Lastly, we show images obtained with a green LED to allow for enhanced vascular detection similar to that of the high-end system, in this representative image of a biopsy confirmed normal cervix with several Nabothian cysts (Fig 10). Note in Fig 10B and 10D the prominence of vasculature indicated by the black arrows in both systems’ green field imaging, with the clear and yellow sacs are the cysts. Again, the POCkeT Colposcope systems provided comparable image quality in pilot *in vivo* imaging to commercial digital colposcope under white field illumination.

## Discussion

Our POCkeT Colposcope platform has comparable optical imaging capabilities to commercial colposcopes as assessed by quantitative image quality metrics using standard imaging targets, simulated gynecologic mannequin, and preliminary *in vivo* imaging of the human cervix. At a working distance of 30 mm, both POCkeT Colposcope generations were able to resolve features sized 27.86 and 24.8 micron thickness, for 2.0MP and 5.0MP POCkeT Colposcope, respectively. This approaches the 22.0-micron thickness resolving power of the Leisegang Optik 2 Digital Colposcope at 7.5X magnification. Additionally our POCkeT Colposcope systems provide sufficient field of view at our target magnifications (diagonal field of view of at least 30–35 mm) and make full use of the imaging frame, unlike the other low cost colposcopy devices, which have limited magnifying capability. Both POCkeT Colposcope systems can provide a FWHM beam diameter of >40 mm to adequately illuminate the cervix and have sufficient optical power when compared to current clinical systems. Other low cost digital colposcopy systems (Apple iPhone 5S) have much larger FWHM beam diameters (~70 mm) and are not as uniform as that of the POCkeT Colposcope platform as seen in the irregular shape of the luminance contours, which could lead to erroneous interpretation of the cervical images. Our findings are in agreement with prior literature in regards to the optical safety of colposcope sources [40].

The POCkeT Colposcope systems have a small amount of lens distortion but are similar in performance to other low cost digital colposcopy systems, at <5% for the 2.0MP system and 1.14% for the 5.0MP system. This is a potential concern as the pixel to length ratio is no longer constant and size and shape of structures will be more distorted at the edges of the image when compared to the center for the 2.0MP POCkeT Colposcope. Margins, size, shape, and border of lesions at the edges of the cervical images are at risk with profound lens distortion leading to potential misinterpretation. We believe we can enhance the images further with post processing of captured images to remove the lens distortion using a derived lens distortion correction coefficient, scale factor, and image processing software like Picture Window Pro [25, 29] and remove regions of specular reflection by using a registration method to correlate multiple images capture of the same cervix and/or a neighborhood averaging method to replace saturated pixels with usable information [41, 42].

The accurate color reproduction of the lesion and the normal tissue is important in the classification when using VIA/VIAM (e.g. Reid’s Colposcopic Index [43] and Swede Score [44]), as there is the potential for erroneous color reproduction to reduce performance of clinical interpreted images from any digital colposcopy system. Standardizing the color reproduction of the cervical images from colposcopes in a system-independent color space like CIELAB would remedy differences due to differential illumination strategies and to reduce the potential variability of interpretation from images capture across different systems [26], which have done our best to implement in our system. Using the Adobe Photoshop “color match” feature, we can minimize the mean square error between system values and the ideal values, which can be applied when post processing to ensure comparable color reproduction across different systems [45]. A potential limitation is that the monitors used by collaborating clinicians to review the cervical images will differ in color reproduction, intensity, and other factors. Our current strategy is for all users to use a standardized viewing device that will also be NIST calibrated (e.g. Google Nexus 7 tablet and/or Nexus 5 smartphone which have high definition resolution of at least 1920x1080, and a display pixel density of > 300 per in). We are also actively investigating an improved color matching technique that we can implement in a near real-time manner.

Furthermore, we have developed an encrypted digital database from the concordant images collected with the high-end digital colposcope and our system using the Research Electronic Data Capture (REDcap) platform. We are getting expert physicians’ interpretations of the images and hope to use this randomized de-identified database to provide online health worker training in colposcopy based screening of pre-cancer/cancer lesion detection and allow for telemedicine-based interpretation of captured images by partner clinician experts. The mobile health app is being developed to interface with this database and includes modules for patient education and health worker job aids.

We are actively prototyping a speculum free mechanism for visualizing the cervix that we can modularly added to our existing prototype. This system would help support and steady the probe and deploy internally to open up the vaginal walls in front of the probe and not from the entrance to the cervix to enhance patient comfort and ease of use. Our rationale for the development of a speculum free approach is that the speculum, which is an added cost, requires sterilization before every use and the number of women that can be screened depends on the number of speculums available and/or the sterilization time. The implementation of the screening program itself can also be a barrier from an adoption perspective. A study in rural Moshi Tanzania of 354 women revealed that key factors for cervical cancer screening were husband approval, level of education, significant concerns about embarrassment and pain due to screening from the speculum, gender of the health provider, and distance to the screening center [46]. In a survey conducted in rural Mexico by the Stanford University School of Medicine, the most frequent reason for not having a cervical exam was anxiety regarding physical privacy. Less frequent reasons were lack of knowledge and difficulty accessing health care [47]. Even in the U.S., where there is greater access to health care, compliance rates with cervical screening vary, and embarrassment and fear of pain during examination have been reported as barriers to screening [48–50]. The speculum itself is a cause of discomfort particularly for women with vaginismus, where there is involuntary tightening of the vagina that is often a result of sexual abuse [51]. East African countries, such as Tanzania have among the highest sexual violence rates worldwide [52, 53]. There is an undeniable relationship between sexual violence and the contraction of HPV and thus it is these women who are in greatest need for frequent cervical screening.

## Supporting Information

S1 File A compressed collection of files that have been internally subdivided into folders that include raw image files, CAD (computer aided drawings) of the custom circuit boards and 3D models for the probe, a detailed bill of materials, and raw spectra for illumination characterization of each system. A caption document for each sub-folder and file (**S1 Detailed File Key**) is found within the archive. Folder (**S2**) contains the set of raw images from quantitative imaging characterization between systems. Folder (**S3**) contains the CAD files for the custom circuit boards used in our POCkeT Colposcope. Folder (**S4**) contains the 3D CAD files for the probe handle and bill of materials. Folder (**S5**) contains the complete set of n = 5 replicate spectra files captured from each digital colposcope system.
(ZIP)
