# Supplementary material for: Design of a Novel Low Cost Point of Care Tampon (POCkeT) Colposcope for Use in Resource Limited Settings
Source: PLoS One. 2015 Sep 2;10(9):e0135869. doi: 10.1371/journal.pone.0135869 (PMC4557989; doi:10.1371/journal.pone.0135869)
Supplement: S1 File — A caption document for each sub-folder and file (S1 Detailed File Key) is found within the archive. Folder (S2) contains the set of raw images from quantitative imaging characterization between systems. Folder (S3) contains the CAD files for the custom circuit boards used in our POCkeT Colposcope. Folder (S4) contains the 3D CAD files for the probe handle and bill of materials. Folder (S5) contains the complete set of n = 5 replicate spectra files captured from each digital colposcope system. (ZIP) [file pone.0135869.s001.zip › S1 Supplementary Files/S1 Detailed File Key.docx]

**Supporting Information Captions**

**S2 Figure. Collection of 3 figures used in performance characterization of various digital colposcopy systems.**  Luminance contour plots for the for each digital colposcope system are plotted with intensity normalized from 0 to 1, with scale bars = 10 mm (**S2 Fig A**), with (from left to right) Leisegang Optik 2, POCkeT Colposcope 5MP (labeled here as 5.0MP TVDC), POCkeT Colposcope 2MP (labeled here as 2.0MP TVDC), Canon SX50HS, and Apple iPhone 5S. Representative images captured of checkerboard targets used to quantify level of distortion are shown in (**S2 Fig B**), with (from left to right) Leisegang Optik 2, POCkeT Colposcope 5MP (labeled here as 5.0MP TVDC), POCkeT Colposcope 2MP (labeled here as 2.0MP TVDC), Canon SX50HS, and Apple iPhone 5S. Representative images captured of a Thorlabs’s USAF1951 resolution target (R3L3S1N) used to determine field of view and resolving power are shown in (**S2 Fig C**), (from left to right) Leisegang Optik 2, POCkeT Colposcope 5MP (labeled here as 5.0MP TVDC), POCkeT Colposcope 2MP (labeled here as 2.0MP TVDC), Apple iPhone 5S, and Canon SX50HS.

**S2 Fig A.tiff**

**S2 Fig B.tiff**

**S2 Fig C.tiff**

**S3 File. Collection of 2 CAD (computer aided design) drawings for the custom PCB (printed circuit boards) used for the POCkeT Colposcope.** The design file (*.*pcb) for the concentric illumination ring for white and green SMD (surface mount device) miniature LEDs (light emitting diodes) (**S3 CAD A**) that can be open with ExpressPCB’s free circuit design package. A black and white screen capture of the concentric ring design can be seen in (**S3 Fig A**). The design file (*.*pcb) (**S3 CAD B**) for the Arduino microcontroller controlled constant current LED drivers that can be opened with ExpressPCB’s free circuit design package. A black and white image of the control board can be seen in (**S3 Fig B**).

**S3 CAD A.pcb**

**S3 CAD B.pcb**

**S3 Fig A.bmp**

**S3 Fig B.bmp**

**S4 File. Collection of five 3 dimensional (3D) CAD (computer aided design) drawings for the custom ABS 3D printed handle for the POCkeT Colposcope and detailed Bill of Material Table.** The design file (*.*) for the polarizer and LED mount (**S4 CAD A**) can be opened with most open source 3D CAD packages. The design file (*.*stl) rear-handle left half shell (**S4 CAD B**) and rear-handle right half shell (**S4 CAD C**) can be opened with most open source 3D CAD packages. The design file (*.*stl) front-handle left half shell (**S4 CAD D**) and front-handle right half shell (**S4 CAD E**) can be opened with most open source 3D CAD packages. The build units for all these files **S4 CAD A to E** are in millimeters and were designed on Dassult Systemes’ SolidWork 2013. These were rapid prototyped on the Stratasys Dimension 1200es ABS fused deposition modeling (FDM). (**S4 Table A**) is the detailed bill of materials for our prototype build.

**S4 CAD A.stl**

**S4 CAD B.stl**

**S4 CAD C.stl**

**S4 CAD D.stl**

**S4 CAD E.stl**

**S4 Table A.docx**

**S5 File. Collection of raw spectra files for all the digital colposcopy illumination systems evaluated.** The dark reference repeated measure (n=5) spectra files were captured at 10 ms integration time (**S5 Spectra DA to DE**) and at 100 ms integration time (**S5** **Spectra DDA to DDE**) are in (*.*.txt) form with wavelength intensity measured from 178 to 888 nm. The white LED illumination of the Leisegang Optik 2 colposcope’s repeated measure (n=5) spectra files were captured at 10 ms integration time (**S5** **Spectra LO2-W-A to E**) and at also at 10 ms integration time for the green filter mode of illumination (**S5** **Spectra LO2-G-A to E**) are in (*.*.txt) form with wavelength intensity measured from 178 to 888 nm. The white halogen illumination of the Wallach Zoomscope colposcope’s repeated measure (n=5) spectra files were captured at 100 ms integration time (**S5** **Spectra WZ-W-A to E**) and at also at 100 ms integration time for the green filter mode of illumination (**S5** **Spectra WZ-G-A to E**) are in (*.*.txt) form with wavelength intensity measured from 178 to 888 nm. The white LED illumination of the 5MP POCkeT Colposcope’s repeated measure (n=5) spectra files were captured at 100 ms integration time (**S5** **Spectra PC-FIVE-W-A to E**) and at also at 100 ms integration time for the 5MP POCkeT Colposcope’s green LED illumination (**S5** **Spectra PC-FIVE-G-A to E**) are in (*.*.txt) form with wavelength intensity measured from 178 to 888 nm. The white LED illumination of the 2MP POCkeT Colposcope’s repeated measure (n=5) spectra files were captured at 100 ms integration time (**S5** **Spectra PC-TWO-W-A to E**) . are in (*.*.txt) form with wavelength intensity measured from 178 to 888 nm.

**S5 Spectra DDA.txt**

**S5 Spectra DDB.txt**

**S5 Spectra DDC.txt**

**S5 Spectra DDD.txt**

**S5 Spectra DDE.txt**

**S5 Spectra DA.txt**

**S5 Spectra DB.txt**

**S5 Spectra DC.txt**

**S5 Spectra DD.txt**

**S5 Spectra DE.txt**

**S5 Spectra LO2-G-A.txt**

**S5 Spectra LO2-G-B.txt**

**S5 Spectra LO2-G-C.txt**

**S5 Spectra LO2-G-D.txt**

**S5 Spectra LO2-G-E.txt**

**S5 Spectra LO2-W-A.txt**

**S5 Spectra LO2-W-B.txt**

**S5 Spectra LO2-W-C.txt**

**S5 Spectra LO2-W-D.txt**

**S5 Spectra LO2-W-E.txt**

**S5 Spectra WZ-G-A.txt**

**S5 Spectra WZ-G-B.txt**

**S5 Spectra WZ-G-C.txt**

**S5 Spectra WZ-G-D.txt**

**S5 Spectra WZ-G-E.txt**

**S5 Spectra WZ-W-A.txt**

**S5 Spectra WZ-W-B.txt**

**S5 Spectra WZ-W-C.txt**

**S5 Spectra WZ-W-D.txt**

**S5 Spectra WZ-W-E.txt**

**S5 Spectra PC-FIVE-G-A.txt**

**S5 Spectra PC-FIVE -G-B.txt**

**S5 Spectra PC-FIVE -G-C.txt**

**S5 Spectra PC-FIVE -G-D.txt**

**S5 Spectra PC-FIVE -G-E.txt**

**S5 Spectra PC-FIVE -W-A.txt**

**S5 Spectra PC-FIVE -W-B.txt**

**S5 Spectra PC-FIVE -W-C.txt**

**S5 Spectra PC-FIVE -W-D.txt**

**S5 Spectra PC-FIVE -W-E.txt**

**S5 Spectra PC-TWO-W-A.txt**

**S5 Spectra PC-TWO-W-B.txt**

**S5 Spectra PC-TWO-W-C.txt**

**S5 Spectra PC-TWO-W-D.txt**

**S5 Spectra PC-TWO-W-E.txt**
