# Supplementary material for: Design of a Novel Low Cost Point of Care Tampon (POCkeT) Colposcope for Use in Resource Limited Settings
Source: PLoS One. 2015 Sep 2;10(9):e0135869. doi: 10.1371/journal.pone.0135869 (PMC4557989; doi:10.1371/journal.pone.0135869)
Supplement: S1 File — A caption document for each sub-folder and file (S1 Detailed File Key) is found within the archive. Folder (S2) contains the set of raw images from quantitative imaging characterization between systems. Folder (S3) contains the CAD files for the custom circuit boards used in our POCkeT Colposcope. Folder (S4) contains the 3D CAD files for the probe handle and bill of materials. Folder (S5) contains the complete set of n = 5 replicate spectra files captured from each digital colposcope system. (ZIP) [file pone.0135869.s001.zip › S1 Supplementary Files/S4/S4 Table A.docx]

**Table 1:** Bill of Materials for the POCkeT Colposcope with estimated cost per unit fabrication cost in US dollars

| Schematic  Label | Component | Manufacturer | Part # | QTY | Function |
| --- | --- | --- | --- | --- | --- |
| 1a | 2.0MP Color CMOS Detector with manual focus only | Vividia | [UM07](http://www.oasisscientific.com/vitiny-um07-usb-digital-endoscope.html) | 1 | Digital Camera Module with USB 1.0 |
| 1b | 5.0MP Color CMOS Detector with Autofocus | Supereyes | [A005+](http://www.supereyes.cc/product/show/query/11.html) | 1 | Digital Camera Module with USB 2.0 |
| 2-1  2-2 | 3D Printed ABS TVDC Case and Handle | Custom,  [Statasys Dimensions 1200es](http://www.stratasys.com/3d-printers/design-series/dimension-1200es#specifications) | | 2 | Ergonomic handle for user and protective casing |
| 3 | 3D Printed ABS Polarizer Mount | Custom,  [Statasys Dimensions 1200es](http://www.stratasys.com/3d-printers/design-series/dimension-1200es#specifications) | | 1 | Spacer to hold detector side polarizer |
| 4 | Linear Glass Polarizer | Edmund Optics | [43-782](http://www.edmundoptics.com/optics/polarizers/linear-polarizers/linear-glass-polarizing-filters/43782/) | 1 | Glare reduction from cervix |
| 5 | Concentric Ring PCB | Custom, [ExpressPCB](http://www.expresspcb.com/) | | 1 | Mounting base for LEDs and Polarizers |
| 6-1 | White 5000K Miniature LEDs | Philips Lumileds | [LXZ1-5070](http://www.mouser.com/ProductDetail/Lumileds/LXZ1-5070/?qs=sGAEpiMZZMt82OzCyDsLFFEWaHNtmc1tFs5coxjKQFw%3d) | 4 | White Field Illumination |
| 6-2 | Green Miniature LEDs | Philips Lumileds | [LXZ1-PM01](http://www.mouser.com/ProductDetail/Lumileds/LXZ1-PM01/?qs=sGAEpiMZZMt82OzCyDsLFJ6%252bvA%2f%252bvM4%2fEzxG4dmJL10%3d) | 4 | Green Field Illumination |
| 7 | High Contrast Linear Polarizer Film, 0.18 mm Thickness | Edmund Optics | [86-183](http://www.edmundoptics.com/optics/polarizers/linear-polarizers/high-contrast-linear-polarizing-film/86183/) | 1 | Glared reduction from cervix |
| 8 | Medical Grade USP Class VI Heat Shrink Wrap Tubing with repeated sterilization compatibility | Insultab | [Xtra-Shield HS-714](http://www.medicalheat-shrinktubing.com/Xtra-Shield-HS-714-Non-Shrink.html) | 1 | Waterproof, chemical resistance barrier; ETO, gamma, autoclave, H_2_O_2_ gas, glutaraldehyde compatible |
| 9 | Micro USB to USB OTG Host Adapter M/F Cable | Startech | [UUSBOTG](http://www.startech.com/Cables/USB-2.0/USB-Adapters/5in-Micro-USB-to-USB-OTG-Host-Adapter-Male-to-Female~UUSBOTG) | 1 | Interfaces camera module with smartphone |
| 10 | Microcontroller | Arduino | [Micro](http://www.mouser.com/ProductDetail/Arduino/A000053/?qs=sGAEpiMZZMvq007EO%252bXAYZAVvNFL4IMQ) | 1 | Microcontroller |
| 11 | System Controller, LED Drivers PCB | Custom, [ExpressPCB](http://www.expresspcb.com/) | | 1 | IC mounting and interface board |
| 12 | Darlington Transistor with TTL interface | Texas Instruments | [ULN2803-APG](http://www.mouser.com/ProductDetail/Toshiba/ULN2803APGCNHZN/?qs=sGAEpiMZZMsn4IaorHFpMPD0aBY1kl0Xme82FfrptBU%3d) | 1 | Allows for PWM control of constant current drivers |
| 13-1  13-2 | Linear Temperature Compensated Constant Current LED Driver ICs | Microchip Inc. | [CL2N3-G](http://www.mouser.com/ProductDetail/Microchip-Technology/CL2N3-G/?qs=sGAEpiMZZMsE420DPIasPsmbGP%2fDsdbv7CfbaiDKVaw%3d)  [CL25N3-G](http://www.mouser.com/ProductDetail/Microchip-Technology/CL25N3-G/?qs=sGAEpiMZZMvtAekNgeAQePMcZCbneqDu) | 3  3 | Constant current drivers for LEDs |
| 14 | 12.8V Rechargeable Lithium Battery Pack | Powerizer | [2061](http://www.batteryspace.com/lifepo414505battery128v600mah4x145054sflat06aratewithpcb.aspx) | 1 | Provides system power for mobile operation |
|  |  |  |  | | |
| 15 | Nexus 5 16GB | Google | [Nexus 5](http://www.google.com/nexus/5/) | 1 | Android based smartphone for image storage and transmission |
