# Supplementary figures and images for: Design of a Novel Low Cost Point of Care Tampon (POCkeT) Colposcope for Use in Resource Limited Settings
Source: PLoS One. 2015 Sep 2;10(9):e0135869. doi: 10.1371/journal.pone.0135869 (PMC4557989; doi:10.1371/journal.pone.0135869)

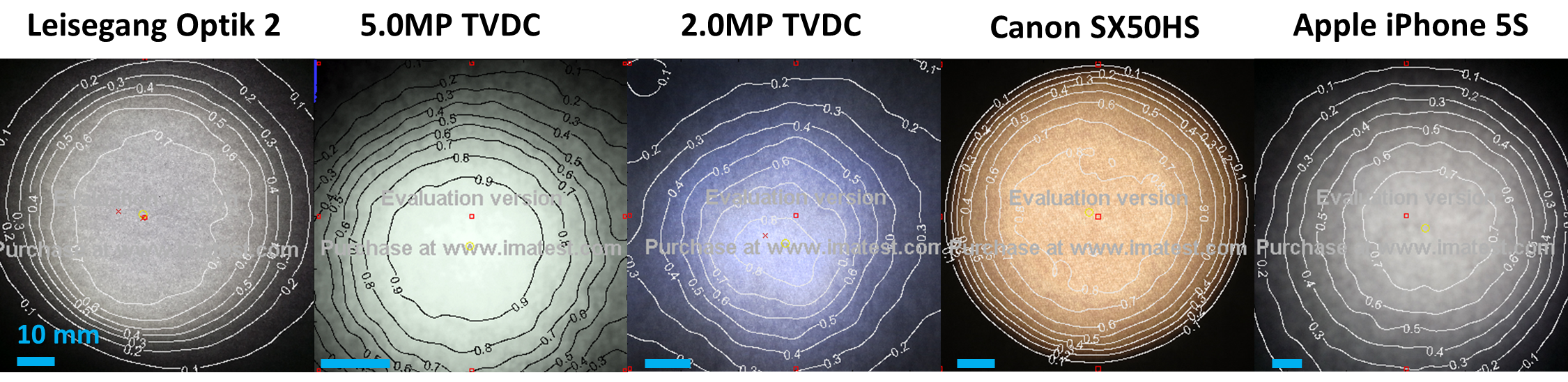

Supplement: S1 File — A caption document for each sub-folder and file (S1 Detailed File Key) is found within the archive. Folder (S2) contains the set of raw images from quantitative imaging characterization between systems. Folder (S3) contains the CAD files for the custom circuit boards used in our POCkeT Colposcope. Folder (S4) contains the 3D CAD files for the probe handle and bill of materials. Folder (S5) contains the complete set of n = 5 replicate spectra files captured from each digital colposcope system. (ZIP) [file pone.0135869.s001.zip › S1 Supplementary Files/S2/S2 Fig A.tif]

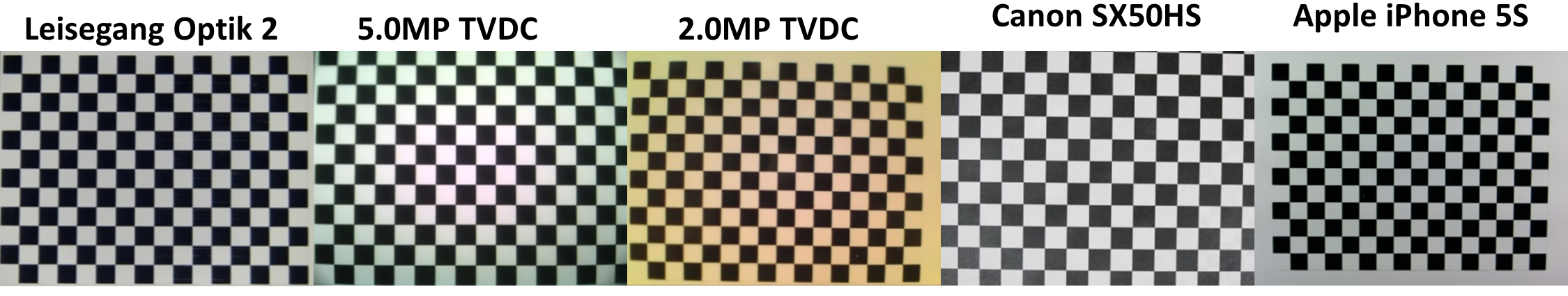

Supplement: S1 File — A caption document for each sub-folder and file (S1 Detailed File Key) is found within the archive. Folder (S2) contains the set of raw images from quantitative imaging characterization between systems. Folder (S3) contains the CAD files for the custom circuit boards used in our POCkeT Colposcope. Folder (S4) contains the 3D CAD files for the probe handle and bill of materials. Folder (S5) contains the complete set of n = 5 replicate spectra files captured from each digital colposcope system. (ZIP) [file pone.0135869.s001.zip › S1 Supplementary Files/S2/S2 Fig B.tif]

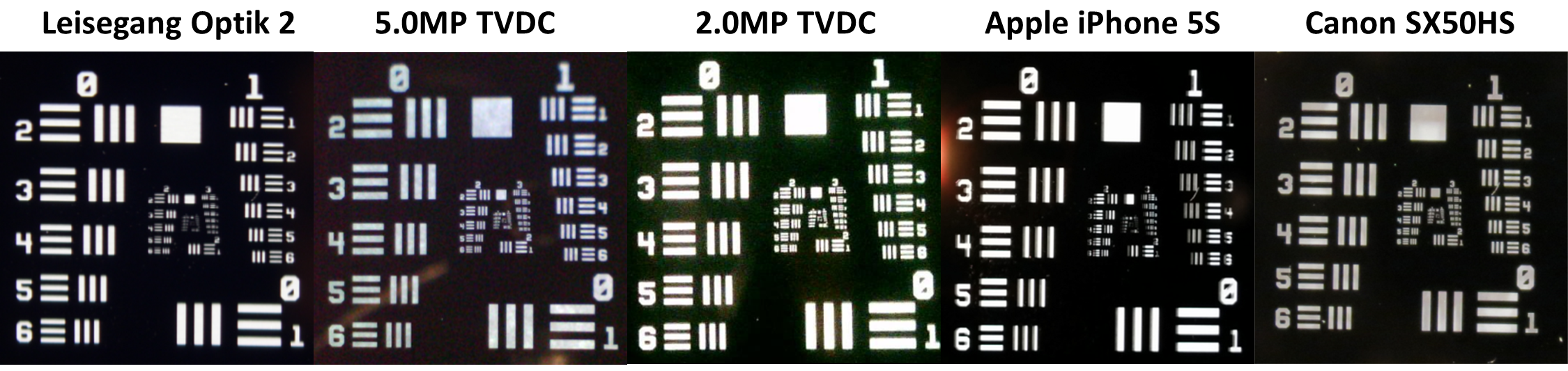

Supplement: S1 File — A caption document for each sub-folder and file (S1 Detailed File Key) is found within the archive. Folder (S2) contains the set of raw images from quantitative imaging characterization between systems. Folder (S3) contains the CAD files for the custom circuit boards used in our POCkeT Colposcope. Folder (S4) contains the 3D CAD files for the probe handle and bill of materials. Folder (S5) contains the complete set of n = 5 replicate spectra files captured from each digital colposcope system. (ZIP) [file pone.0135869.s001.zip › S1 Supplementary Files/S2/S2 Fig C.tif]

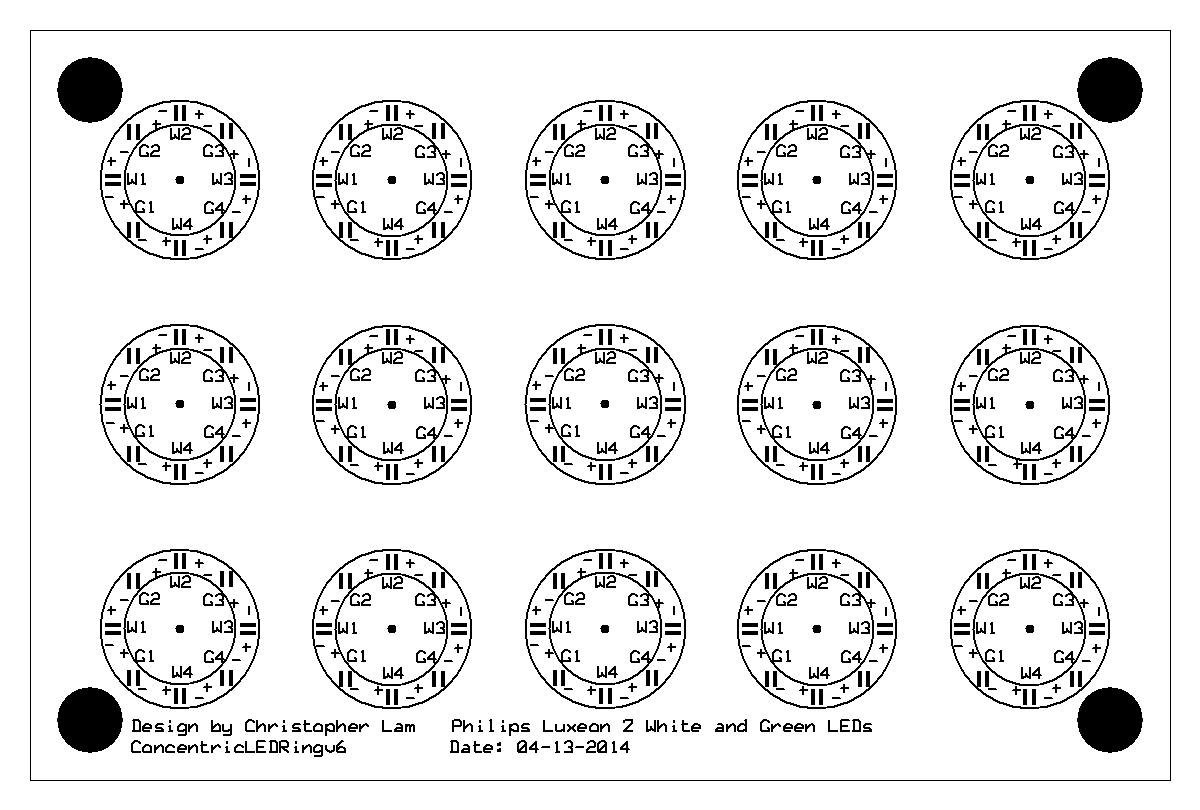

Supplement: S1 File — A caption document for each sub-folder and file (S1 Detailed File Key) is found within the archive. Folder (S2) contains the set of raw images from quantitative imaging characterization between systems. Folder (S3) contains the CAD files for the custom circuit boards used in our POCkeT Colposcope. Folder (S4) contains the 3D CAD files for the probe handle and bill of materials. Folder (S5) contains the complete set of n = 5 replicate spectra files captured from each digital colposcope system. (ZIP) [file pone.0135869.s001.zip › S1 Supplementary Files/S3/S3 Fig A.bmp]

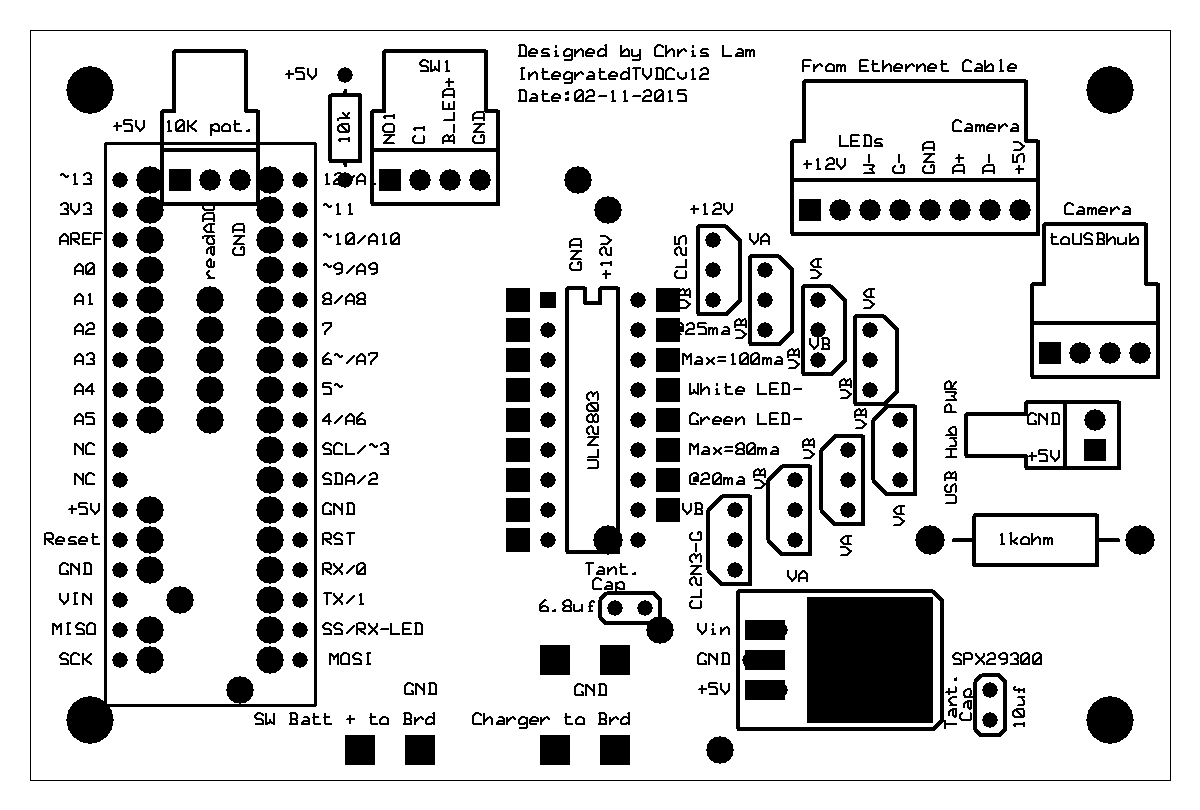

Supplement: S1 File — A caption document for each sub-folder and file (S1 Detailed File Key) is found within the archive. Folder (S2) contains the set of raw images from quantitative imaging characterization between systems. Folder (S3) contains the CAD files for the custom circuit boards used in our POCkeT Colposcope. Folder (S4) contains the 3D CAD files for the probe handle and bill of materials. Folder (S5) contains the complete set of n = 5 replicate spectra files captured from each digital colposcope system. (ZIP) [file pone.0135869.s001.zip › S1 Supplementary Files/S3/S3 Fig B.bmp]
